# Immunological Significance of Prognostic DNA Methylation Sites in Hepatocellular Carcinoma

**DOI:** 10.3389/fmolb.2021.683240

**Published:** 2021-05-26

**Authors:** Qianhui Xu, Yuanbo Hu, Shaohuai Chen, Yulun Zhu, Siwei Li, Feng Shen, Yifan Guo, Tao Sun, Xiaoyu Chen, Jinpeng Jiang, Wen Huang

**Affiliations:** ^1^ The Second Affiliated Hospital and Yuying Children’s Hospital of Wenzhou Medical University, Wenzhou, China; ^2^ Zhejiang University School of Medicine, Hangzhou, China

**Keywords:** DNA methylation sites, hepatocellular carcinoma, prognosis, tumor immune environment, immune checkpoint blockade, tumor mutation burden

## Abstract

**Background:** Hepatocellular carcinoma (HCC) is a tumor with high morbidity and high mortality worldwide. DNA methylation, one of the most common epigenetic changes, might serve a vital regulatory role in cancer.

**Methods:** To identify categories based on DNA methylation data, consensus clustering was employed. The risk signature was yielded by systematic bioinformatics analyses based on the remarkably methylated CpG sites of cluster 1. Kaplan–Meier analysis, variable regression analysis, and ROC curve analysis were further conducted to validate the prognosis predictive ability of risk signature. Gene set enrichment analysis (GSEA) was performed for functional annotation. To uncover the context of tumor immune microenvironment (TIME) of HCC, we employed the ssGSEA algorithm and CIBERSORT method and performed TIMER database exploration and single-cell RNA sequencing analysis. Additionally, quantitative real-time polymerase chain reaction was employed to determine the LRRC41 expression and preliminarily explore the latent role of LRRC41 in prognostic prediction. Finally, mutation data were analyzed by employing the “maftools” package to delineate the tumor mutation burden (TMB).

**Results:** HCC samples were assigned into seven subtypes with different overall survival and methylation levels based on 5′-cytosine-phosphate-guanine-3′ (CpG) sites. The risk prognostic signature including two candidate genes (LRRC41 and KIAA1429) exhibited robust prognostic predictive accuracy, which was validated in the external testing cohort. Then, the risk score was significantly correlated with the TIME and immune checkpoint blockade (ICB)–related genes. Besides, a prognostic nomogram based on the risk score and clinical stage presented powerful prognostic ability. Additionally, LRRC41 with prognostic value was corroborated to be closely associated with TIME characterization in both expression and methylation levels. Subsequently, the correlation regulatory network uncovered the potential targets of LRRC41 and KIAA1429. Finally, the methylation level of KIAA1429 was correlated with gene mutation status.

**Conclusion:** In summary, this is the first to identify HCC samples into distinct clusters according to DNA methylation and yield the CpG-based prognostic signature and quantitative nomogram to precisely predict prognosis. And the pivotal player of DNA methylation of genes in the TIME and TMB status was explored, contributing to clinical decision-making and personalized prognosis monitoring of HCC.

## Introduction

As one of the aggressive malignancies, hepatocellular carcinoma (HCC) is characterized by high morbidity rate and low survival rate in the world (Bray et al., 2018; Forner et al., 2018; Yang et al., 2019). And tumor stages at diagnosis significantly affected the prognosis of HCC patients (Liu et al., 2016). The clinicopathological staging and treatment system—the Barcelona Clinic Liver Cancer (BCLC) algorithm—was the most extensively applied classification method for patients with HCC (Faria et al., 2014; Couri and Pillai, 2019). Given HCC was of high heterogeneity, patients exhibited distinct clinical outcomes of treatment and different survival times even in the same stage (Miao et al., 2014; Forner et al., 2018; Zhang et al., 2019). Owing to higher precision and less side effects, immune checkpoint blockade (ICB) therapy has brought much benefit to various patients in a wide range of tumors. Approximately one-fifth of patients responded to ICB treatment according to preclinical trial results, suggesting immune checkpoint inhibitor administration may be a potential treatment for HCC patients (Cheng et al., 2019). Besides, the contrasting outcome of immune checkpoint blockade (ICB) therapy in distinct population and multiple malignant tumors has focused light on the characterization of tumor immune microenvironment (TIME) (El-Khoueiry et al., 2017). The complexity of the tumor immune microenvironment may be a pivotal player in tumor immune evasion and responses to clinical treatment of HCC (Finkin et al., 2015; Hackl et al., 2016). Anson M. et al. pointed out that type I NKT cells presented enrichment in HCC and played a protective role against tumors (Anson et al., 2012). Not only infiltrating CD4^+^ CTLs but also circulating CD4^+^ CTLs were independent predictive indicators of OS and DFS in patients with HCC (Fu et al., 2013). Therefore, further exploration of HCC biological mechanisms and underlying molecular processes, with also the discovery of vital novel indicators for prognosis prediction and outcome of clinical therapy, is the task of top priority.

DNA methylation, a well-characterized genetic alteration, refers to the transfer of the methylated group of S-adenosyl-L-methionine (SAM) to the pyrimidine ring of cytosine residues on DNA (Jaenisch and Bird, 2003; Jin and Liu, 2018; Pfeifer, 2018). The methylation process often occurs in the cytosine of 5′-cytosine-phosphate-guanine-3′ (CpG) structure and is mediated by DNA methyltransferase (DNMT) (Moore et al., 2013). DNA hypermethylation can repress the targeted gene expression level, further mediating the transcriptional regulation of biological processes including DNA repair, cell cycles, and tumor progression, suggesting its crucial player in accelerating malignancies (Lea et al., 2018; Luo et al., 2018; Pfeifer, 2018; Eyvazi et al., 2020). Abnormal methylation changes (i.e., anti-tumor oncogene hypermethylation and oncogene hypomethylation) are regarded as important events in tumorigenesis (Huang et al., 2011; Lambert et al., 2011; Yang et al., 2017). Published researches pointed out that hypomethylation of CpG may result in the initiation of oncogene; meanwhile, CpG dinucleotide hypermethylation can cause anti-oncogene silencing (Antequera and Bird, 1993). Many studies have supported strong evidence to support that the clinical utility, such as early diagnostic indicators or prognostic predictive biomarkers, of these changes in multiple cancers (Fu, 2015; Klutstein et al., 2016; Pfeifer, 2018; Ebrahimi et al., 2020). For HCC, Villanueva A et al. developed a methylation-based risk signature to precisely predict the prognosis of HCC patients (Villanueva et al., 2015). Based on three CpGs, Qiu J et al. generated a methylation risk signature to accurately predict recurrence in early-stage HCC patients (Qiu et al., 2017). Hence, it is of significant and encouraging value for prognostic prediction and clinical intervention of patients with HCC to yield an efficient signature on the basis of differential DNA methylation.

Researches focusing on the correlation of DNA methylation with diagnosis and prognosis of HCC have been extensively explored (Villanueva et al., 2015; Xu et al., 2017); however, systematic analysis to establish a robust and novel prognostic signature that could predict prognosis and outcome was rarely reported.

Herein, to facilitate identifying promising subtypes to precisely subdivide HCC patients, we addressed the HCC classification system *via* screening prognostic subtypes on the basis of methylation data of HCC cases. Furthermore, we analyzed DNA methylation profiles of HCC patients from the TCGA databank, in order to filter the prognostic methylation biomolecular factors and generate the risk signature and prognostic nomogram, contributing to new insight into the TIME context and immunotherapy outcome of HCC. Besides, our risk signature may provide guidance for doctors to perform prognosis estimation and individualized clinical intervention, realizing further precision treatment to enhance therapeutic efficacy accordingly.

## Methods

### Data Selection and Preprocessing

Transcript data from 378 HCC patients were downloaded from the TCGA database (https://portal.gdc.cancer.gov/). After excluding cases without complete information, clinical profiles of 240 patients were downloaded and are shown in Supplementary Table S1. DNA methylation information was obtained from the UCSC Cancer Browser (https://xena.ucsc.edu/). 430 DNA methylation data and annotation files were downloaded from the Illumina Human Methylation 450 platform (https://xenabrowser.net/datapages/?dataset=TCGA-LIHC.methylation450.tsv&host=https%3A%2F%2Fgdc.xenahubs.net&removeHub=https%3A%2F%2Fxena.treehouse.gi.ucsc.edu%3A443) for further study. The threshold for rejection of the CpG sites is given below: The site was missing in more than 70% of the samples; it was located in the sex chromosomes and single-nucleotide polymorphisms; CpGs above 2 kb upstream to 0.5 kb downstream; and cross-reactive genome CpG sites (Chen et al., 2013). Ultimately, we employed 226 HCC samples with both expression matrix and methylation data for further analysis. We obtained fragments per thousand base million (FPKM) of HCC patients from the TCGA database and converted the FPKM value to the transcript per million (TPM) value. The LIRI-JP dataset including 260 HCC samples from the ICGC database was employed as the external validation group. The corresponding expression profiling information and the clinical data (Supplementary Table S2) were downloaded from the ICGC database (https://dcc.icgc.org). Four categories of somatic mutation data of HCC samples were obtained from The Cancer Genome Atlas (TCGA) portal. We singled out the mutation files that were obtained through the “SomaticSniper variant aggregation and masking” platform for subsequent analysis. We prepared the Mutation Annotation Format (MAF) of somatic variants and implemented the “maftools” (Mayakonda et al., 2018) R package.

### Determination of Classification Features for Methylation Sites

In order to classify prognostic-related HCC molecular subtypes, the methylation level of each CpG site, age, gender, clinicopathological stage, tumor status (T, M, and N), and survival data were employed to construct univariate Cox proportional risk regression models. Supplementary Table S3 presents 610 significant CpG sites (*p* < 0.05). Then, these significant sites were introduced into multivariate Cox proportional risk regression models. Finally, we selected the CpG sites that were still significant as the classification features (*p* < 0.05, Supplementary Table S4) that can significantly affect the prognosis of HCC patients.

### Identification of Prognostic Methylation Subtypes Using Consensus Clustering

In order to define the HCC subgroups based on the most variable methylated CpG sites (Supplementary Table S5), consensus clustering was conducted using the ConcensusClusterPlus R package (Wilkerson and Hayes, 2010). Each case we collated was partitioned into k groups on the basis of a user-specified clustering algorithm (k-means). This process was repeated for a user-specified number of repetitions, offering a method of establishing consensus values and estimating the stability of the established clustering. Pairwise consensus values, referring to “the proportion of clustering runs in which two subjects gathered together,” were calculated and stored in a consensus matrix for each k. The final results from agglomerative hierarchical consensus clustering based on one-Pearson correlation distances were also divided into k groups. The exported graphs contained the consensus matrices, consensus cumulative distribution function (CDF) plot, delta area plot, and tracking plot. We determined the k value if there were a low relative change in the area under the CDF curve, relatively high conformity in the clusters, and a low variation coefficient. The pheatmap package in R was used to create the heatmap (Diao et al., 2018).

### Correlation Between the Subtypes and Prognosis or Clinical Characteristics in HCC

To explore the overall survival of HCC patients whose subgroups were defined from DNA methylation information, we employed the “survival” package (Huang et al., 2017; Li et al., 2019; Xiong et al., 2019), and the results are illustrated in Kaplan–Meier plots. The log-rank test was used to identify the significant differences among different clusters. To determine the distinction in categorical profiles as the clinical variables among different subtypes, the chi-squared test was analyzed. All tests were two-sided with a significance level of 0.05.

### Functional Annotation and Enrichment Analysis

To further reveal the CpG site–related gene expression pattern in the standpoint of fundamental biology, Kyoto Encyclopedia of Genes and Genomes (KEGG) and gene ontology (GO) enrichment analyses on CpG site–related genes significantly affected prognosis. We employed gene set enrichment analysis (GSEA) to explore underlying mechanisms correlated with the prognostic signature. The gene sets of “c2. cp.kegg.v7.2. symbols.gmt [Curated]” from the Molecular Signatures Database were analyzed through gene set enrichment analysis (GSEA) (Subramanian et al., 2005) with a Java program (http://software.broadinstitute.org/gsea/index.jsp).

### Identification of Prognostic Risk Signature

Two hundred twenty-six HCC samples were randomly divided into the discovery and validation groups at the ratio of 1:1 for integrated analysis utilizing the “caret” package. Both discovery and validation groups were required to meet the following criteria (Forner et al., 2018): samples were randomly assigned to discovery and validation cohorts (Bray et al., 2018) and the clinical characteristics of subjects in these groups were similar (Supplementary Table S6). The validation group with 112 cases and the whole cohort were used to validate results obtained from the discovery set. The TCGA cohort was randomly classified into discovery and validation groups again to demonstrate the repeatability of the risk model. The detailed clinical variables of samples are recorded in Supplementary Table S7. The “survival” package with coxph function was employed to create a Cox proportional hazards model based on the combination of expression profiles for remarkably methylated CpG sites corresponding to genes in cluster 1 (Supplementary Table S8) and follow-up data (Zhang, 2016; Bhattacharjee et al., 2020). The candidate methylation-related genes significantly affect prognosis (*p* < 0.05), which were identified by employing a univariate proportional hazards model of the expression level of seven methylation-related genes. Next, the risk coefficient of each gene was obtained by performing the LASSO regression algorithm with the “glment” package after the deletion of highly correlated genes. Finally, two genes were selected and introduced into a prognostic predictive signature in HCC. The risk score of each patient was calculated by the following equation: risk score = sum of risk coefficients * methylation-related gene expression level.

### Validation of Prognostic Risk Signature

Each patient obtained the risk score according to the above formula together with their clinical data. Based on the median risk score, patients were classified into low/high‐risk subgroups for further study. Kaplan–Meier survival curves were analyzed to compare prognosis of different subgroups. We then performed the time-dependent receiver-operating characteristic (ROC) curve analysis to assess the prognostic value, which was achieved by comparing the specificity and sensitivity in predicting prognosis on the basis of the risk score. Furthermore, multivariate Cox regression was employed to demonstrate the risk score was an independent prognostic indicator for HCC patients. The predictive performance of the as-constructed risk signature was then confirmed in the validation group (*n* = 112) and combined cohort. Additionally, the ICGC cohort was employed as an external validation group to facilitate extensive application. Each test was two-sided, and *p* < 0.05 was deemed statistically significant.

### Construction of Prognostic Nomogram

To comprehensively assess the prognosis predictive ability of risk signature, stage, gender, age, and WHO grade for one/two/three-year OS, time-dependent receiver-operating characteristic (ROC) curves were performed to calculate the area under the curve (AUC) values (Blanche et al., 2013). To contribute to a quantitative manner predicting the overall survival of patients with HCC, we established a nomogram containing the risk score and other clinical variables to estimate one‐, two‐, and three‐year overall survival possibility. Subsequently, we analyzed the calibration curve that shows the prognostic value of the as-constructed nomogram.

### Landscape of Tumor Immune Environment

To better identify the difference of TIME characteristics between different subgroups, we performed several analyses as follows. Firstly, the “GSEABase” R package with regard to 29 immunity-related signatures was used to further reveal distinction of TIME characterization between different subgroups. Then, the R package “CIBERSORT” was performed to calculate 22 immune cell subpopulations in HCC. Subsequently, immune infiltration information consists of every specimen’s immune cell fraction (i.e., B cells, CD4^+^ T cells, CD8^+^ T cells, dendritic cells, macrophages, and neutrophils) downloaded from tumor immune estimation resource (TIMER) (https://cistrome.shinyapps.io/timer/).

### Immunotherapeutic Significance of Prognostic Signature and LRRC41

Referring to existing studies, it was found that the expression level of immune checkpoint blockade–related key genes might be correlated with the clinical outcome of immune checkpoint inhibitor blockade treatment^42^. Herein, we employed six key genes of immune checkpoint blockade therapy: programmed death ligand 1 (PD‐L1, also known as CD274), programmed death 1 (PD‐1, also known as PDCD1), programmed death ligand 2 (PD‐L2, also known as PDCD1LG2), T‐cell immunoglobulin domain and mucin domain–containing molecule‐3 (TIM‐3, also known as HAVCR2), indoleamine 2,3‐dioxygenase 1 (IDO1), and cytotoxic T‐lymphocyte antigen 4 (CTLA‐4) in HCC (43–45). To elucidate the potential player of the as-constructed risk signature in ICB treatment of HCC, we correlated the prognostic signature and the expression level of six immune checkpoint blockade key genes (i.e., IDO1, CTLA-4, HAVCR2, CD274, PDCD1, and PDCD1LG2). Subsequently, we systematically determined the expression value of 47 immune checkpoint blockade–related genes (i.e., PDCD1) between patients from different subgroups to investigate the potential role of the risk score in ICB treatment.

### Analysis of the Distribution of LRRC41 in HCC by Single-Cell RNA Sequencing

To further explore the role of LRRC41 in TIME, we employed single-cell transcriptome sequencing data from GSE140228 (Zhang et al., 2019), which are the transcriptome data of CD45^+^ immune cells made by the Zemin Zhang team for HCC patients. The researchers uploaded the hepatic carcinoma single-cell RNA sequencing data of the study to an interactive website (http://cancer-pku.cn:3838/HCC/) to facilitate the researcher’s in-depth exploration of related fields. Herein, we use 10× Genomics sequencing data to analyze the expression of LRRC41 in tumor, hepatic lymph node, adjacent liver, ascites, and blood and compare the abundance of LRRC41 in immune cell subsets in tumor tissues.

### Calculation of Tumor Mutational Burden

TMB was defined as the number of somatic, coding, base replacement, and insertion–deletion mutations per megabase of the genome examined using non-synonymous and code-shifting indels under a 5% detection limit. TMB scores for each sample were calculated through dividing the number of somatic mutations by the total length of exons (38 million). The R package “maftools” (Mayakonda et al., 2018) was used to calculate the total number of somatic non-synonymous point mutations within each sample.

### Experimental Validation

L02 (human hepatic cell line) and two human HCC cell lines (MHCC-97H cells and HCC-LM3 cells) were purchased from the Cell Bank of the Type Culture Collection of the Chinese Academy of Sciences, Shanghai Institute of Biochemistry and Cell Biology. The cell lines were all cultured in Dulbecco’s minimum essential media (DMEM) plus 10% fetal bovine serum (FBS; Invitrogen, Carlsbad, CA, United States). All cell lines were grown without antibiotics in a humidified atmosphere of 5% CO_2_ and 99% relative humidity at 37°C. Three different cell lines were subjected to quantitative real-time polymerase chain reaction (qRT-PCR).

### RNA Isolation and qRT-PCR Analysis

Total RNA was extracted from cells using TRIzol (Invitrogen, Carlsbad, CA, United States) according to provided instructions. RNA concentration and purity were measured in triplicates utilizing the NanoDrop 2000 spectrophotometer (Thermo Scientific Inc., Waltham, MA, United States). Then, total RNA was reverse transcribed to cDNA using the cDNA Reverse Transcription Kit (Vazyme, Nanjing, China). To determine the expression of LRRC41, cDNAs were subjected to qRT-PCR using SYBR Green Real-Time PCR Master Mix (Takara) in Applied Biosystems 7500/7500 Fast Real-Time PCR System (Thermo Fisher Scientific). All samples were analyzed in triplicates. Glyceraldehyde-3-phosphate dehydrogenase (GAPDH) levels were used as the endogenous control, and the relative expression of LRRC41 was calculated using the 2-ΔΔCt method. The sequences of primers used for PCR were as follows: LRRC41, 5′- TGG​CTG​GCG​AGA​AGG​AGG​ATG -3′ (forward) and 5′- CAA​GGT​GGA​GAT​GCT​GCG​GAA​TC -3′ (reverse), and GAPDH, 5′-CAG​GAG​GCA​TTG​CTG​ATG​AT-3′ (forward) and 5′-GAA​GGC​TGG​GGC​TCA​TTT-3′ (reverse).

### Statistics

The correlation between data with a normal distribution was analyzed with Pearson’s correlation analysis. Comparisons of two groups were made with the t-test; if there were more than two groups, the comparison was made with the analysis of variance (ANOVA) test. The Wilcox test was used to compare the methylation levels between the seven clusters. Samples with a CIBERSORT output value of *p* < 0.05 were screened for further analysis (Chen et al., 2018). All the statistical analysis results described below were obtained with the R software. The significance level was set to *p* < 0.05.

## Results

### Determination of Potential Prognostic DNA CpG Sites

After methylation data were obtained from the TCGA cohort and pretreated as described above, we screened 19392 CpG sites among which 610 methylation sites were determined as candidate DNA methylation indicators for prognostic prediction in HCC patients *via* univariate Cox regression analysis (Supplementary Table S3). A subsequent multivariate Cox regression analysis was employed to identify these CpG sites closely correlated with prognosis. And 136 sites were identified and deemed potential prognosis-related CpG sites (Supplementary Table S4).

### Consensus Clustering to Identify Subgroups of HCC and Inter-Cluster Prognosis Analysis

**FIGURE 1:**
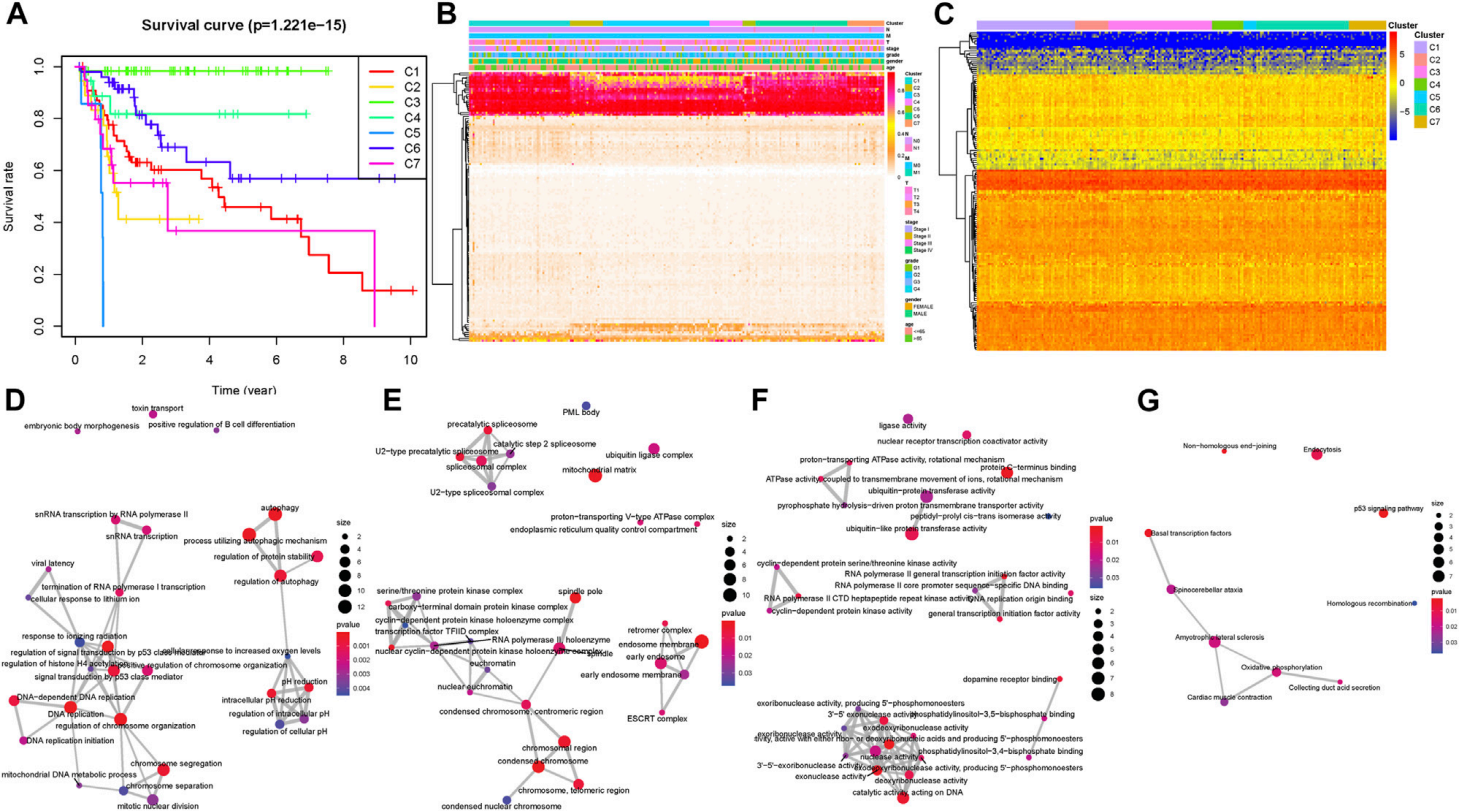
Inter-cluster prognosis and clinical significance analysis. **(A)** Survival curves of different tumor subtypes. The number of samples in each cluster is shown in parentheses in the legend. The log-rank test was used to assess the statistical significance of differences between subtypes. **(B)** Heatmap of the DNA methylation levels in the seven clusters and their clinical characteristics. The color from red to white shows a trend from high expression to low expression. **(C)** Cluster analysis heatmap for annotated genes associated with the 184 CpG sites. Crosstalk analysis of prognostic DNA methylation genes in the enriched KEGG pathway gene ontology **(D–F)** and KEGG **(G)**.

Taking advantage of consensus clustering, we found that when k = 7, there is high consistency in these clusters with a relatively low coefficient of change but no appreciable variation in the area under the CDF curve and delta area (Supplementary Figures S1A,B). The heatmap of the seven clusters with a good polymerization effect was mostly diagonal (Supplementary Figure S1C). Kaplan–Meier analysis indicated there was a remarkable distinction among different clusters (*p* = 1.221e−15), suggesting that clustering results classified the patients into seven categories with distinct overall survival (Figure 1A). The distribution of clinical characteristics (age, gender, historical staging, T status, N status, and M status) in each subtype is presented in Figure 1B. Subsequently, the expression level of the CpG site–related genes was determined in the subtypes. Figure 1C presents the heatmap of gene expression values. To better comprehend the methylation site expression from the viewpoint of biology level, KEGG and GO enrichment analyses on CpG site–related genes were employed. Functional annotation results indicated that overexpressed methylation-related genes were primarily enriched in autophagy, endosome membrane, protein C−terminus binding, and p53 signaling pathway (Figures 1D–G, corresponding GO and KEGG, respectively). Subsequently, we analyzed intra-cluster fraction for the seven clusters based on clinical characteristics (Supplementary Figures S2A–G, age, gender, stage, and TNM category, respectively). Tendencies for correlations between clinical parameters and categories were as follows: clusters 1, 4, 6, and 7 with higher clinical grade; clusters 1 and 5 with advanced stage; clusters 4, 5, and 7 with higher T status; clusters 5, 6, and 7 with higher N status; and clusters 1 and 6 with higher M status. These results suggest that each clinical variable correlated with a distinct intra-cluster distribution.

### Inter-Cluster Difference of Methylation Levels

**FIGURE 2:**
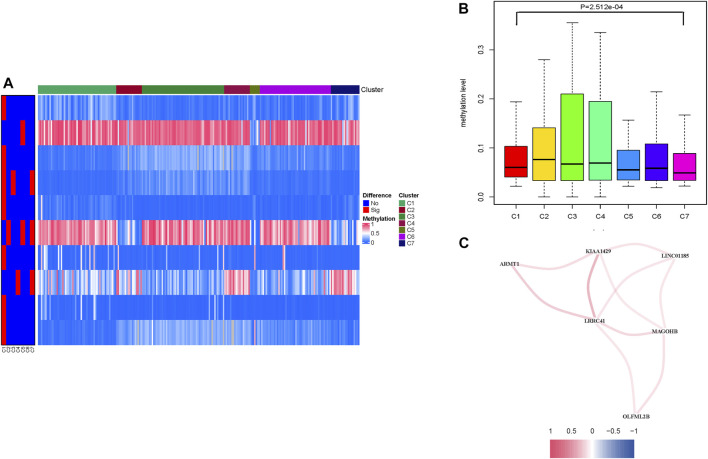
Levels of prognosis-related DNA methylation sites of different HCC clusters. **(A)** Heatmap of abnormally expressed methylation sites in the seven subtypes. The heat rectangle on the left map: red represents statistically significant difference, whereas blue represents no significance. **(B)** Comparison of the methylation levels among different clusters. **(C)** Correlation analysis between methylation candidate genes (LRRC41, KIAA1429, LINC01185, MAGOHB, OLFML2B, and ARMT1). Red represents positive correlation, while blue represents negative correlation.

One hundred thirty-six prognostic methylation sites’ methylation levels are shown in Supplementary Table S5. A heatmap presents distinction of methylation levels between selected clusters and the other clusters (Figure 2A). The heatmap result shows that the significantly changed CpG sites were mostly distributed in cluster 1. Combined with survival rate analysis (Figure 1A), indicating that cluster 1 had a middle survival time among these clusters, we picked the differential CpG sites’ methylation data according to cluster 1 to develop boxplot. A consistent result was obtained in methylation level analysis, which shows that cluster 1 had a middle methylation level among different clusters (Figure 2B). The differential methylated CpG sites’ corresponding genes in cluster 1 were introduced as candidate genes for further study.

### Construction of Prognostic Risk Signature

To further reveal the potential interaction between these candidate methylation genes, correlation was employed to comprehensively visualize the methylation interaction network (Figure 2C; Supplementary Figure S3A, *p* < 0.05). Notably, LRRC41 was positively and significantly correlated with five hub genes, suggesting its indispensable role in methylation regulation. In order to investigate the prognosis predictive value of differential methylated CpG sites’ corresponding genes from cluster 1 (Supplementary Table S8), univariate Cox regression on candidate genes’ expression profiles was performed, finding two out of seven candidate genes were significantly correlated with overall survival (Supplementary Table S9, *p* < 0.05). Next, the LASSO algorithm was employed to identify methylation-related genes with the most robust prognosis prediction capability (Supplementary Figures S3B,C; Table S10). Finally, the two candidate genes LRRC41 and KIAA1429 were introduced into a methylation-based risk prognostic signature in HCC. The relationship of each methylation-associated gene with overall survival is presented in Supplementary Table S11.

The risk score was obtained as follows: risk score = (0.1682 ∗ expression value of LRRC41) + (0.0981 ∗ expression value of KIAA1429). Then, each HCC patient together with the corresponding risk score was classified into low/high-risk groups based on the median value.

### Identification of the Prognosis Signature in HCC

**FIGURE 3:**
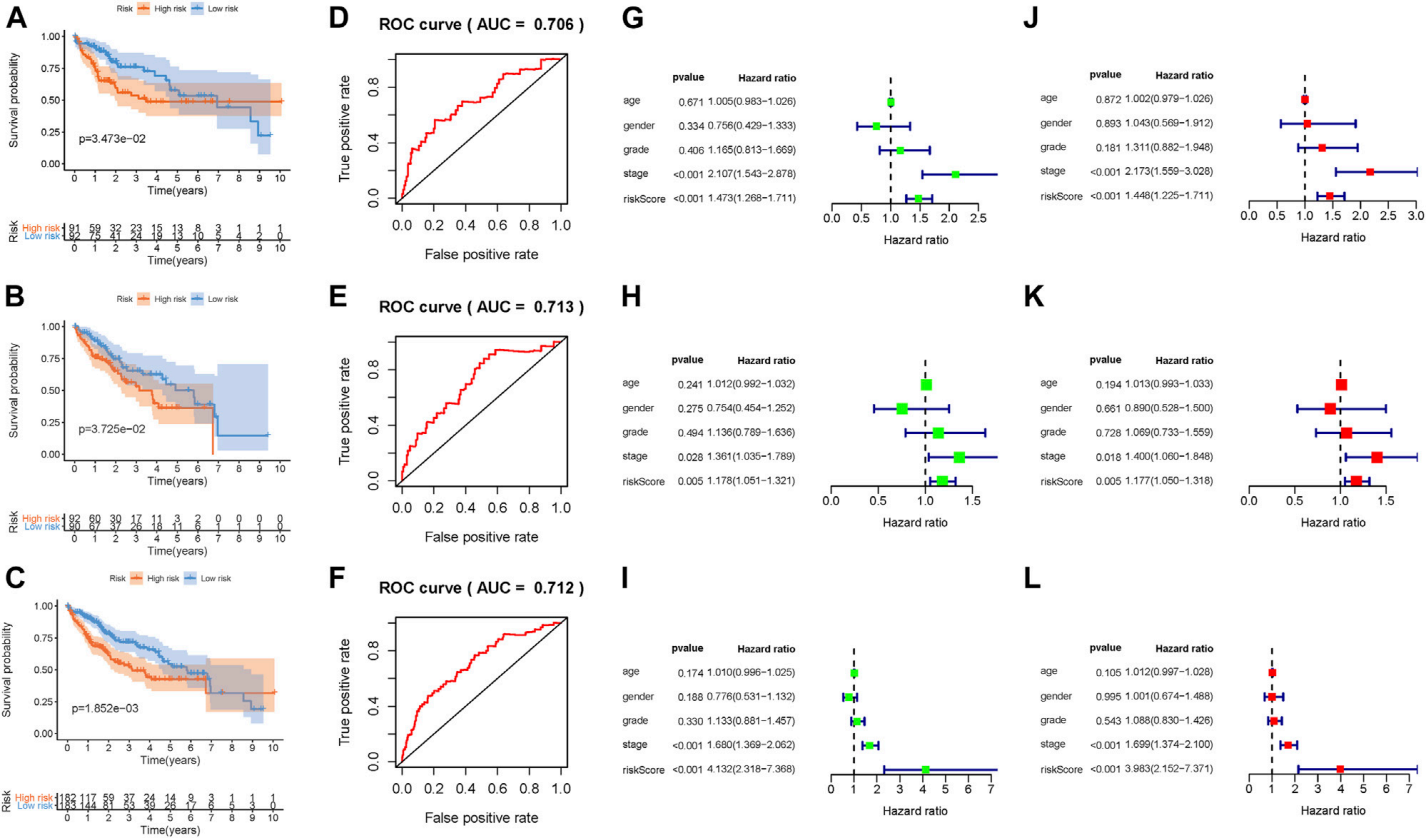
Development of the prognostic risk signature based on DNA methylation. Kaplan–Meier curve analysis presenting difference of overall survival between the high-risk and low-risk groups in the training group **(A)**, testing group **(B)**, and combination cohort **(C)**. ROC curve analysis of the risk scores for overall survival predictive significance in the training group **(D)**, testing group **(E)**, and combination cohort **(F)**. The AUC was calculated for ROC curves, and sensitivity and specificity were calculated to assess score performance. Univariate Cox regression analyses of overall survival in the training group **(G)**, testing group **(H)**, and combination cohort **(I)**. Multivariate Cox regression analyses of overall survival in the training group **(J)**, testing group **(K)**, and combination cohort **(L)**.

Patients in the TCGA-LIHC cohort were randomly divided into the discovery set and validation set (Supplementary Tables S11, S12). The distributions of two methylation-related genes’ expression levels with patients and corresponding groups are displayed in Supplementary Figure S4A. Supplementary Figures S4D,G show that distributions of the risk score and survival status are plotted in, indicating that low-risk HCC patients had better prognosis. The Kaplan–Meier curve further demonstrated that low-risk patients had significantly longer overall survival relative to patients with high risk (*p* = 3.473e-01; Figure 3A). To estimate the specificity and sensitivity of risk signature for differentiating low-risk patients from high-risk patients, we analyzed ROC curve analysis. We observed that the area under curves of prognostic signature at one‐year overall survival was up to 0.706, indicating encouraging efficiency of prognostic performance (Figure 3D). Furthermore, univariate Cox analysis showed that the risk score significantly correlated with overall survival [hazard ratio (HR): 1.473, 95% CI: 1.268–1.711, *p* < 0.001; Figure 3G], and multivariate Cox analysis further presented that the risk score was an independent prognostic factor in HCC (HR: 1.448, 95% CI: 1.225–1.711, *p* < 0.001; Figure 3J).

### Validation of Prognostic Signature for HCC

**FIGURE 4:**
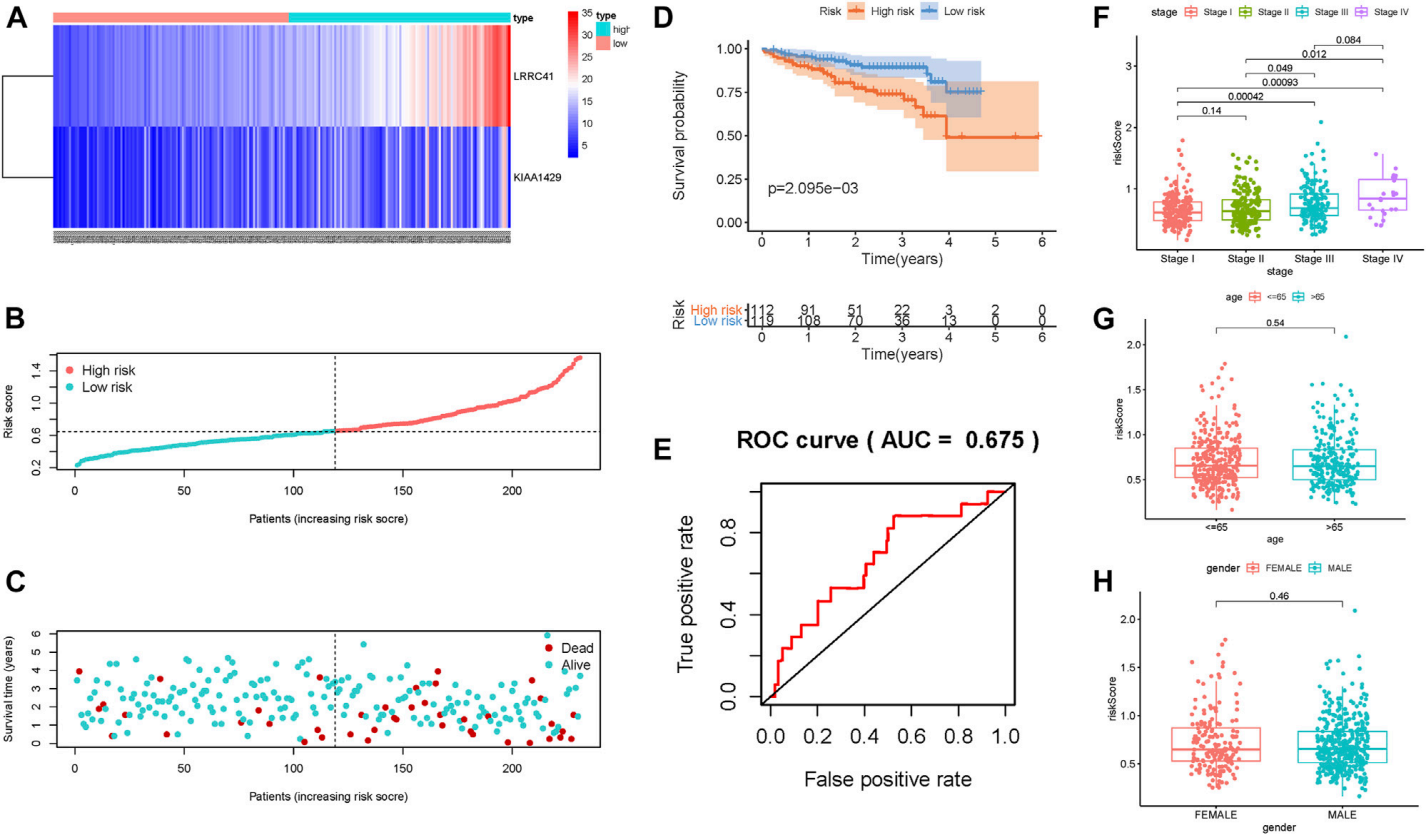
Validation of the risk prognostic signature in the external validation group. **(A)** Heatmap of the candidate gene expression level in HCC. The color from red to blue shows a trend from high expression to low expression. **(B)** Distribution of the DNA methylation–based signature risk score. **(C)** Survival status and interval of HCC patients. **(D)** Kaplan–Meier curve analysis presenting difference of overall survival between the high-risk and low-risk groups. **(E)** ROC curve analysis of the risk scores for overall survival predictive significance. Comparison of risk scores in different subgroups based on age **(F)**, gender **(G)**, and clinicopathological stage **(H)**.

In order to better assess its prognosis predictive accuracy, we examined above results in the validation group and combination cohort. The figures show the distributions of methylation-related genes’ expression levels, survival, and risk scores of patients in the testing set (Supplementary Figures S4B,E,H) and the combination cohort (Supplementary Figures S4C,F,I). The survivorship curve shows that patients with low risk had higher survival probability than high-risk patients in both the validation group (Figure 3B, *p* = 3.725e-02) and the combination cohort (Figure 3C, *p* = 1.852e-03). The value of area under the ROC curve (AUC) was up to 0.713 in the testing set (Figure 3E) and 0.712 in the combined cohort (Figure 3F), indicating strong prognosis predictive power in different groups. Likewise, the risk signature was an independent prognostic indicator significantly correlated with overall survival in both univariable and multivariable regression analyses of the testing set as well as the combined cohort (Figures 3H,I,K,L). Besides, the signature was employed in the LIRI-JP cohort to validate the external prognosis predictive performance. Figures 4A–C show the distributions of genes’ expression levels, risk scores, and survival time in the ICGC validation cohort. Survival analysis (*p* = 2.095e-03; Figure 4D) and ROC curve analysis (AUC = 0.675; Figure 4E) also indicated that this risk score model had an excellent prognosis predictive performance in the LIRI-JP group. Additionally, consistent results were obtained and great prognostic value of the risk model was corroborated in another random classification (Supplementary Figures S5, S6, corresponding training set and testing set, respectively).

The results of correlation of the risk score with clinical variables presented that the advanced clinical stage (most *p* < 0.05, Figure 4F) and risk score remarkably increased. However, there was no significant difference of risk score in age subgroups and gender subgroups (Figures 4G,H).

### Further Confirmation of Prognostic Performance of Signature in HCC

**FIGURE 5:**
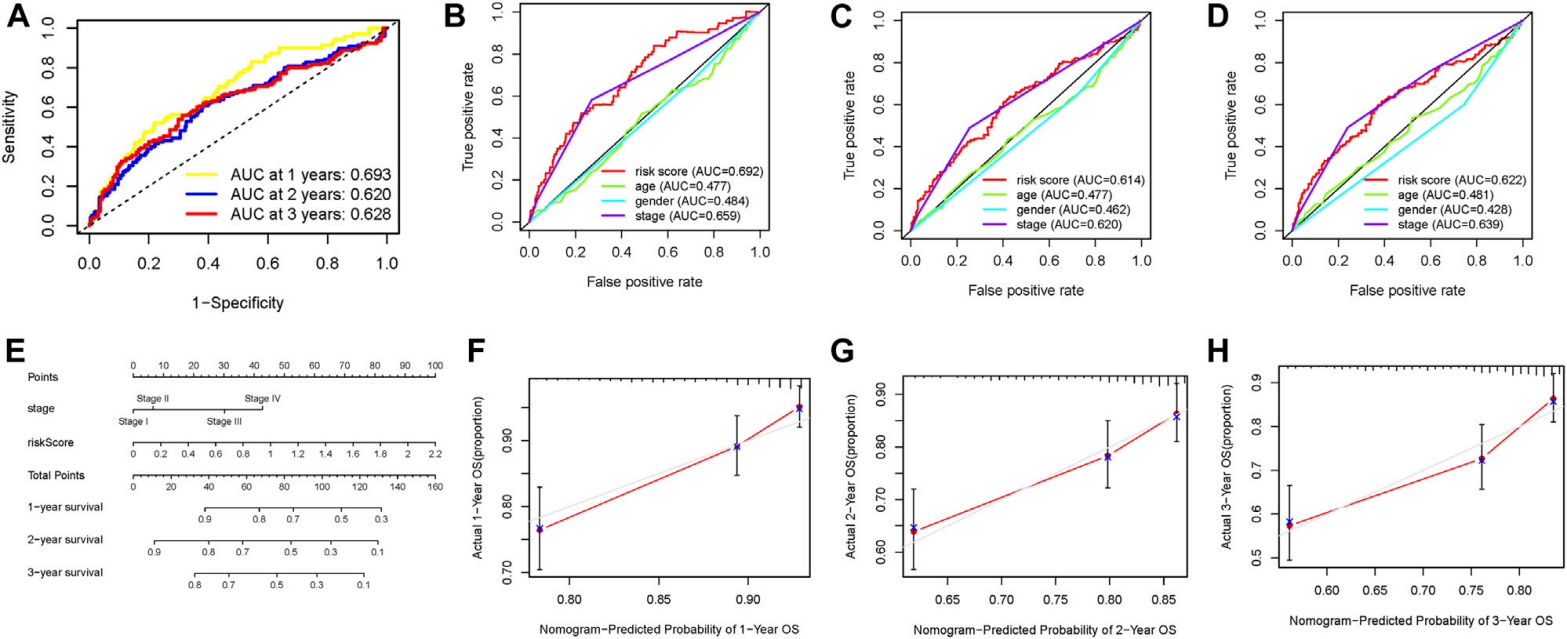
Validation of prognostic efficiency of the DNA methylation–based signature in HCC. **(A)** ROC curve analysis was employed to estimate the prediction value of the prognostic signature. **(B–D)** Areas under curves (AUCs) of the risk scores for predicting one-, two-, and three-year overall survival time with other clinical characteristics. **(E)** Nomogram was assembled by stage and risk signature for predicting the survival of HCC patients. **(F)** One‐year nomogram calibration curves. **(G)** Two‐year nomogram calibration curves. **(H)** Three‐year nomogram calibration curves.

Subsequently, we plotted an ROC analysis curve, and the observed AUC value at one-, two-, and three-year overall survival was 0.693, 0.620, and 0.628, respectively, suggesting excellent prognostic prediction performance (Figure 5A). In order to compare the prognostic predictive efficiency of risk signature with other traditional clinical parameters (age, gender, stage, and grade), we gathered above clinical characteristics and then performed the ROC curve analysis for one-, two-, and three-year survival time and demonstrated that the value of AUC of risk score was the highest (Figures 5B–D). The risk score and clinicopathological stage were introduced into plotting a nomogram to quantitatively predict the overall survival of HCC patients (Figure 5E). Age, gender, and grade were eliminated owing to AUCs lower than 0.55. Calibrated curves demonstrated good prognosis prediction accuracy of one-, two-, and three-year overall survival in the as-constructed nomogram (Figures 5F–H).

Furthermore, to confirm whether prognostic signature remained a robust prognosis prediction validity indicator in patients subdivided into different subtypes according to clinicopathological variables, stratification analysis was performed. Relative to patients with low risk, patients in the high-risk group presented lower survival probability in both the young (<=65) and old (>65) subgroups (Supplementary Figures S7A,B). And the prognostic signature presented a powerful prognostic prediction value in both patients gendered male and female (Supplementary Figures S7C,D) and patients in the early stage (Supplementary Figure S7E), whereas there was no significant difference of overall survival between low/high-risk patients in the advanced stage (Supplementary Figure S7F). These results indicated that the prognostic signature can be a novel and powerful overall survival predictor in HCC.

### Correlation of Prognostic Score With TIME Context in HCC

**FIGURE 6:**
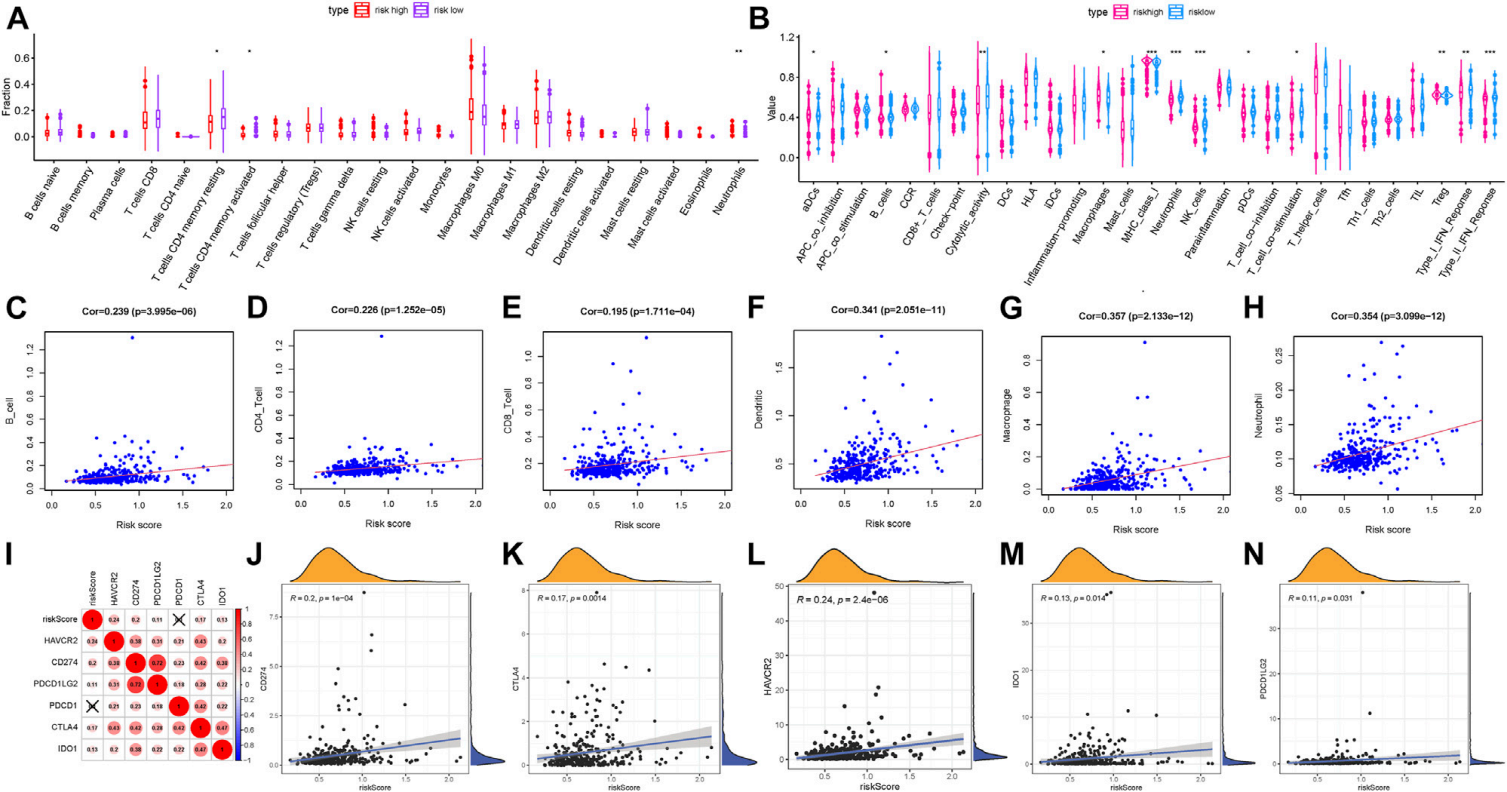
Correlation of the prognostic risk score with TIME characterization of HCC. **(A)** Results of the CIBERSORT algorithm of two risk subgroups. **(B)** Comparison of ssGSEA analysis in two risk score subgroups. **(C)** Relationship between this signature and B cells. **(D)** Relationship between this signature and CD4 T cells. **(E)** Relationship between this signature and CD8 T cells. **(F)** Relationship between this signature and dendritic cells. **(G)** Relationship between this signature and macrophages. **(H)** Relationship between this signature and neutrophils. **(I)** Correlation analysis between immune checkpoint inhibitors (CD274, PDCD1, PDCD1LG2, CTLA4, HAVCR2, and IDO1) and the prognostic risk signature. **(J)** Correlation between the prognostic risk signature and CD274. **(K)** Correlation between the prognostic risk signature and CTLA4. **(L)** Correlation between the prognostic risk signature and HAVCR2. **(M)** Correlation between the prognostic risk signature and IDO1. **(N)** Correlation between the prognostic risk signature and PDCD1LG2.

In order to explore whether the risk score could be a potential indicator of TIME, we analyzed the relationship of the risk score with ssGSEA signatures and TIC proportion and level (assessed using the CIBERSORT algorithm). Firstly, the CIBERSORT results showed that activated memory CD4 T cells were significantly more abundant in high-risk patients, whereas patients with low risk presented higher infiltration of resting memory CD4 T cells (Figure 6A), indicating the phenotype of memory CD4 T cells contributes to the formation of molecular risk to further predict prognosis. Then, the population of aDCs, macrophages, and Tregs and expression of MHC class I were more in the high-risk group; meanwhile, the infiltration of B cells, neutrophils, NK cells, and pDCs, cytolytic activity, T-cell costimulation, and IFN response were higher in the low-risk group (Figure 6B). Furthermore, we analyzed whether the prognostic signature was correlated with immune infiltration. We observed that the risk score had significantly positive correlation with infiltrating B cells (*r* = 0.239; *p* = 3.995e−06), infiltrating CD4 T cells (*r* = 0.226; *p* = 1.252e−05), infiltrating CD8 T cells (*r* = 0.195; *p* = 1.711e−04), infiltrating dendritic cells (*r* = 0.341; *p* = 2.051e−11), infiltrating macrophages (*r* = 0.357; *p* = 2.133e−12), and infiltrating neutrophils (*r* = 0.354; *p* = 3.099e−12; Figures 6C–H).

The above results provided robust evidence to support that the methylation sites’ prognostic signature may act as a vital player to elucidate the context of TIME and further predict clinical immunotherapy efficiency for HCC patients.

### Association of Prognostic Signature With ICB Vital Genes and Immune Infiltration

Subsequently, we correlated six key immune checkpoint inhibitor genes (PDCD1, CD274, PDCD1LG2, CTLA‐4, HAVCR2, and IDO1) (Kim et al., 2017; Nishino et al., 2017; Zhai et al., 2018). And we analyzed the correlation between the prognostic signature and ICB vital genes to explore its potential player in immunotherapy (Figure 6I). We observed that the risk score had significantly positive correlation with CD274 (*r* = 0.2; P = 1e−04; Figure 6J), CTLA4 (r = 0.17; *p* = 0.0014; Figure 6K), HAVCR2 (*r* = 0.24; *p* = 2.4e−06; Figure 6L), IDO1 (r = 0.13; *p* = 0.014; Figure 6M), and PDCD1LG2 (*r* = 0.11; *p* = 0.031; Figure 6N), suggesting the risk prognostic signature might play a crucial role in the monitoring of the ICB therapy outcome in patients with HCC. According to correlation analysis, we observed that 15 of 47 ICB-related genes’ (i.e., CD274) expression levels were remarkably higher in patients with high risk relative to low-risk ones (Supplementary Figure S8A). These results demonstrated that the methylation sites’ risk signature may provide a novel approach to predict immunotherapeutic efficiency in HCC.

### Function Analysis of Risk Signature

To better understand the potential player of the methylation sites’ prognostic signature mediated in the underlying mechanism of HCC, we performed GSEA analysis in not only the high‐risk group but also the low‐risk group. GSEA enrichment analysis results presented that the high-risk score mainly enriched in pathways, including the ERBB signaling pathway, Wnt signaling pathway, mTOR signaling pathway, and VEGF signaling pathway (Supplementary Figure S8B).

### LRRC41 Significantly Affected Overall Survival and Correlates With Immune Infiltration ICB Vital Targets

**FIGURE 7:**
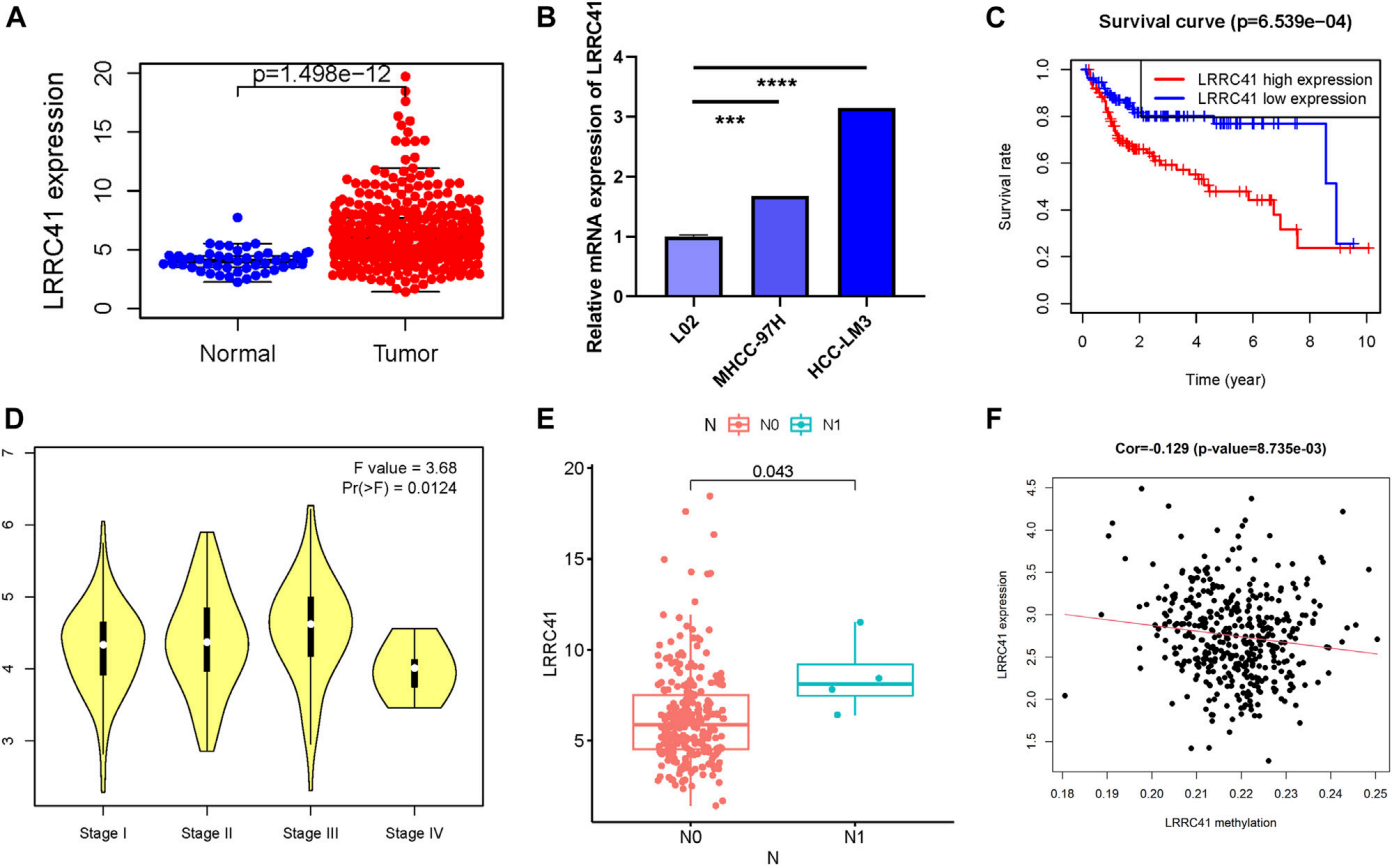
Clinical significance of LRRC41 in HCC. LRRC41 was upregulated in HCC samples based on the TCGA dataset **(A)** and experimental validation **(B)**, and a higher LRRC41 expression level was significantly correlated with poorer prognosis **(C)**. The expression of LRRC41 had significant difference between major pathological stages **(D)** and tumor N category **(E)**. **(F)** The correlation of the LRRC41 expression level with LRRC41 methylation level.

LRRC41 whose expression level was upregulated was the methylation site–related gene and deemed the negative indicator. Thus, the potential player of LRRC41 in HCC was investigated in further validation experiments. Firstly, we determined the expression level of LRRC41 between paracancerous samples and cancer tissues according to the TCGA database. Compared with normal samples, the expression level of LRRC41 was higher in tumor samples (Figure 7A). By using qRT-PCR, we compared the expression level of LRRC41 between two different tumor cell lines and normal liver cell line. In accordance with previous results, LRRC41 was overexpressed in HCC cells compared with hepatic cells (Figure 7B). In order to better evaluate the prognosis predictive ability of LRRC41, survival curve analysis was performed and plotted between LRRC41 high- and low-expressed patients. We found that lower LRRC41 expression significantly suggested higher overall survival probability (Figure 7C, *p* = 6.539e−04). The expression level analysis among major clinical stages showed that LRRC41 was expressed significantly different among distinct clinicopathological stages (Figure 7D, F = 3.68 and *p* = 0.0124). We observed that the higher the N status, the higher the LRRC41 expression level (Figure 7E). Furthermore, the expression level of LRRC41 was significantly and negatively correlated with the methylation level of LRRC41 (Figure 7F, *r* = −0.129, *p* = 8.735e–03).

**FIGURE 8:**
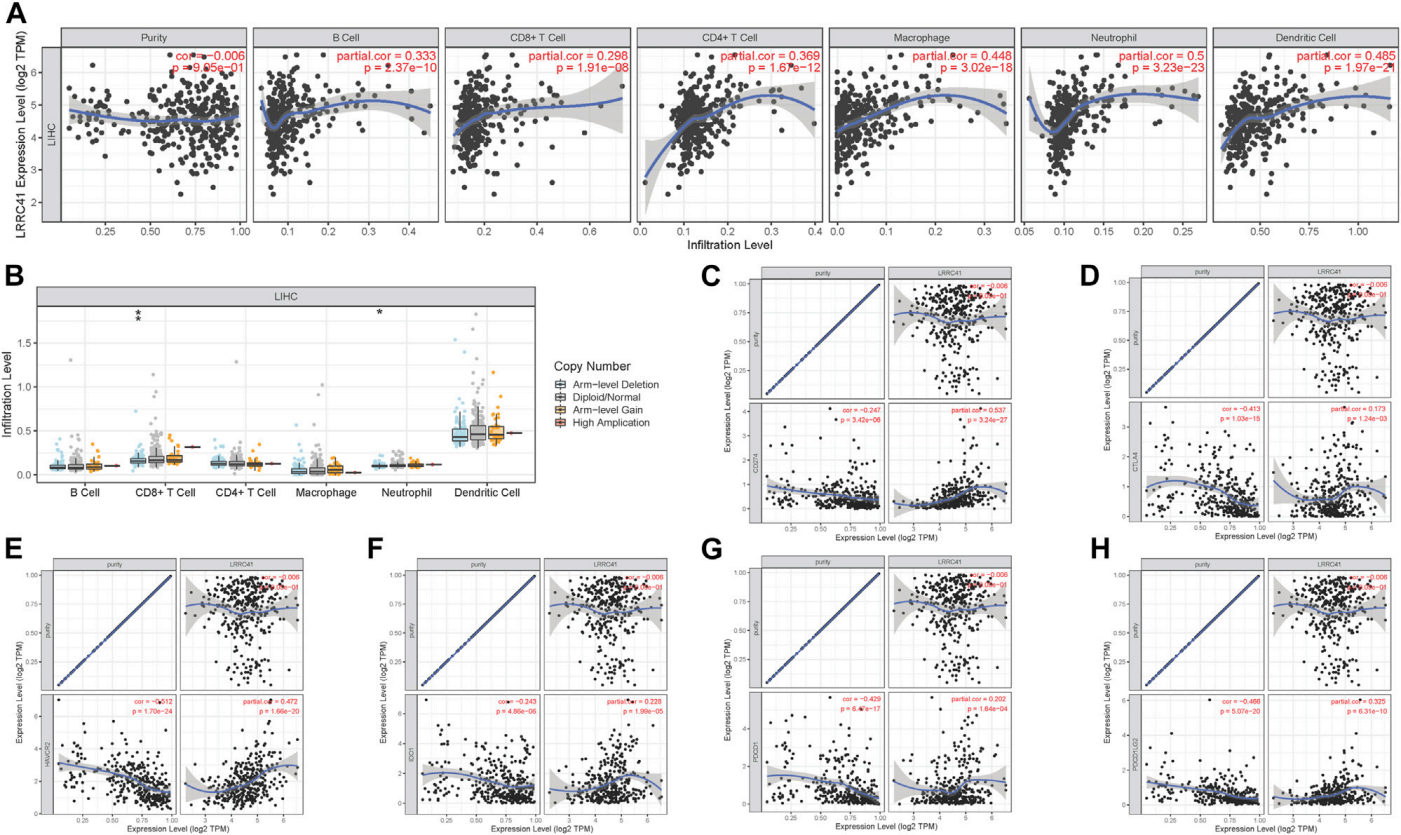
Role of LRRC41 in TIME and immunotherapy of HCC. **(A)** Correlation analysis of LRRC41 expression level with infiltrating B cells, CD4^+^ T cells, CD8^+^ T cells, dendritic cells, macrophages, and neutrophils using TIMER. **(B)** Comparison of tumor infiltration levels among HCC samples with different somatic copy number alterations in LRRC41. The association between the expression levels of LRRC41 and CD274 **(C)**, CTLA4 **(D)**, HAVCR2 **(E)**, IDO1 **(F)**, PDCD1 **(G)**, and PDCD1LG2 **(H)** using TIMER.

To better research the association between the LRRC41 expression level and immune infiltration, we explored the correlation between the expression level of LRRC41 and immune infiltration *via* TIMER. The results indicated that the LRRC41 expression was significantly correlated with B cells (*r* = 0.333; *p* = 2.37e−10), CD8^+^ T cells (*r* = 0.298; *p* = 1.91e−08), CD4^+^ T cells (*r* = 0.369; *p* = 1.67e−12), macrophages (r = 0.448; *p* = 3.02e−18), neutrophils (r = 0.5; *p* = 3.23e−23), and dendritic cells (*r* = 0.485; *p* = 1.97e−21; Figure 8A). Furthermore, distinct mutational types of LRRC41 were correlated with infiltration of neutrophils and CD8^+^ T cells (Figure 8B).

Next, we analyzed the correlation between the LRRC41 expression level and ICB key genes’ expression levels adjusted by tumor purity by TIMER to explore the potential role of LRRC41 in ICB treatment. TIMER results showed that LRRC41 presented significantly positive correlation with CD274 (*r* = 0.537; *p* = 3.24e−27), CTLA4 (*r* = 0.173; *p* = 1.24e−03), HAVCR2 (*r* = 0.472; *p* = 1.66e−20), IDO1 (*r* = 0.228; *p* = 1.99e−05), PDCD1 (*r* = 0.202; *p* = 1.64e−04), and PDCD1LG2 (*r* = 0.325; *p* = 6.31e−10; Figures 8C–H), suggesting LRRC41 has a vital role in ICB therapy.

### Association Between LRRC41 and TIME Characterization

**FIGURE 9:**
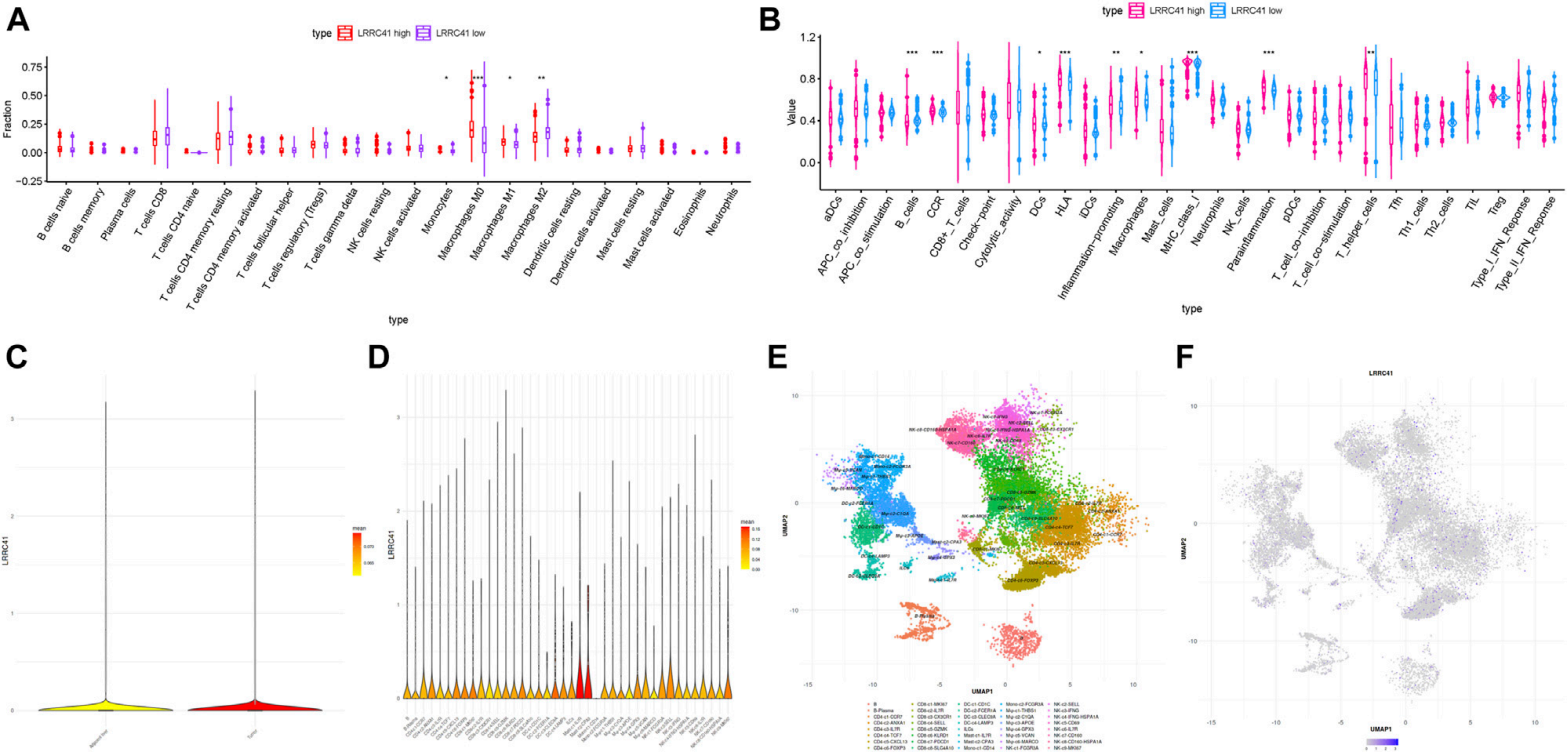
Discrepancy of low and high LRRC41 expression subgroups in terms of TIME characterization. **(A)** Comparison of CIBERSORT results in two LRRC41 expression subgroups. **(B)** Difference of immune-related signatures between low- and high-LRRC41 subgroups. Single-cell RNA sequencing analysis of LRRC41 abundance in various tissues and immune cell subtypes of HCC patients. **(C)** Analysis of the enrichment of LRRC41 in tumor and adjacent liver. **(D)** Analysis of the enrichment of LRRC41 in immune cell subtypes in tumor tissue. **(E)** (Uniform Manifold Approximation and Projection) UMAP map of immune cells in tumor. **(F)** UMAP map of the LRRC41 expression level in tumor tissue.

In order to further reveal the role of LRRC41 in the formation of characteristics in TIME of HCC, we performed correlation analysis of expression value of LRRC41 with ssGSEA enrichment (by the GSEABase method) and immune infiltration fraction and level (using the CIBERSORT algorithm) and further conducted single-cell transcriptome sequencing data analysis. Patients with HCC were divided into low/high-LRRC41 subtypes based on the median value of LRRC41 expression level. The CIBERSORT results presented that the LRRC41 expression level was positively correlated with the abundance of macrophages M0 and M1 whereas negatively correlated with monocytes and macrophage M2 (Figure 9A). According to ssGSEA results, inflammation promotion and parainflammation were activated, CCR and HLA were upregulated, and infiltration of macrophages and T helper cells was remarkably elevated with the LRRC41 expression being increased (Figure 9B). According to the results of single-cell transcriptome sequencing data analysis, we observed that the expression value of LRRC41 is more in tumor tissues than paracancerous tissues (Figure 9C). And LRRC41 is mainly expressed in Mast-c1-IL7R cells and Mast-c2-CPA3 cells in HCC tumor tissues (Figure 9D). Figures 9E,F present the distribution of LRRC41 in immune cells of tumor. Based on the previous findings, mast cell proteases play the crucial role in promoting tumor angiogenesis (de Souza Junior et al., 2015), suggesting LRRC41 may act as a positive player in HCC progression.

**FIGURE 10:**
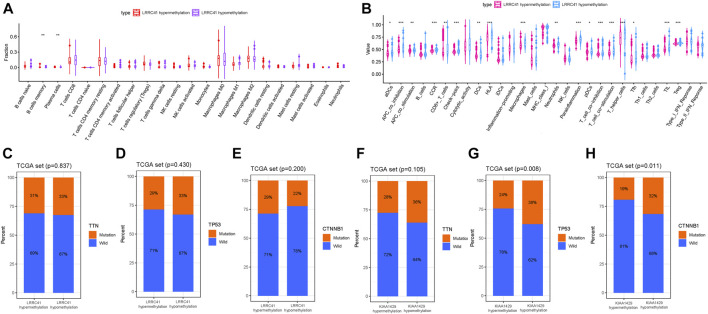
Discrepancy of low and high LRRC41 methylation subgroups in terms of TIME characterization. **(A)** Comparison of CIBERSORT results in two LRRC41 methylation subgroups. **(B)** Difference of immune-related signatures between LRRC41 hypermethylation and LRRC41 hypomethylation subgroups. Correlation of candidate DNA methylation with gene mutated status. **(C)** Correlation of TTN mutation with LRRC41 methylation. **(D)** Correlation of TP53 mutation with LRRC41 methylation. **(E)** Correlation of CTNNB1 mutation with LRRC41 methylation. **(F)** Correlation of TTN mutation with KIAA1429 methylation. **(G)** Correlation of TP53 mutation with KIAA1429 methylation. **(H)** Correlation of CTNNB1 mutation with KIAA1429 methylation.

To further investigate the potential role of the LRRC41 methylation level in TIME characterization, HCC samples were grouped into hypermethylation and hypomethylation subtypes based on the median value of LRRC41 methylation level. The CIBERSORT results pointed out that the infiltration of memory B cells was elevated, but plasma cells were downregulated in the LRRC41 hypermethylation group (Figure 10A).

Additionally, APC costimulation, T-cell costimulation, and parainflammation were activated, Checkpoint, CCR and HLA were upregulated and infiltration of aDCs, CD8^+^ T cells, DCs, macrophages, neutrophils, pDCs, T helper cells, Tfh, TIL, and Treg was remarkably elevated with LRRC41 methylation being decreased (Figure 10B). Collectively, these results suggested that both methylation and expression levels of LRRC41 might be pivotal players in the context of TIME and immune response of HCC.

### Potential Role of KIAA1429 in Prognostic Prediction, Immune Cell Infiltration, and Immunotherapeutic Significance

To further reveal the biological role of KIAA1429 in immune cell infiltration, the correlation of the expression value of KIAA1429 with immune cell infiltration was analyzed *via* TIMER. The results indicated that the KIAA1429 expression was significantly correlated with B cells (*r* = 0.279; *p* = 1.39e−07), CD8^+^ T cells (*r* = 0.121; *p* = 2.54e−02), CD4^+^ T cells (*r* = 0.269; *p* = 4.20e−07), macrophages (*r* = 0.232; *p* = 1.53e−05), neutrophils (*r* = 0.274; *p* = 2.46e−07), and dendritic cells (*r* = 0.278; *p* = 1.85e−07; Supplementary Figure S9A).

Next, the expression value of KIAA1429 in normal tissues and tumor samples was analyzed based on the TCGA database. Relative to normal tissues, KIAA1429 was upregulated in cancer samples (Supplementary Figure S9B). To assess the prognostic value of KIAA1429, the survival curve was analyzed between KIAA1429 high- and low-expressed groups. The result showed that patients with low LRRC41 presented significant advantage of overall survival time (Supplementary Figure S9C, *p* = 0.0039).

Subsequently, the correlation of the KIAA1429 expression level and immunotherapy key genes’ expression levels adjusted by tumor purity by TIMER was analyzed to uncover the potential player of KIAA1429 in immunological treatment. TIMER results showed that KIAA1429 presented significantly positive correlation with CD274 (*r* = 0.226; *p* = 2.23e−05), CTLA4 (*r* = 0.213; *p* = 6.45e−05), HAVCR2 (*r* = 0.264; *p* = 6.29e−07), IDO1 (*r* = 0.117; *p* = 2.99e−02), PDCD1 (*r* = 0.15; *p* = 5.17e−03), and PDCD1 (*r* = 0.141; *p* = 8.90e−03; Supplementary Figures S9D–H), suggesting KIAA1429 has a vital role in immunotherapy.

### Regulatory Network Based on LRRC41 and KIAA1429 in HCC

To further explore the biological mechanism of methylation regulation, correlation networks based on LRRC41 and KIAA1429 were constructed, respectively. A total of 47 interactors were identified in the LRRC41-based network (Supplementary Figure S10A), while 186 interactors were determined in the KIAA1429-based network (Supplementary Figure S10B). We reviewed the literature correlated with these interactors in HCC, CUL5 (Ma et al., 2013), RNF7 (Yu et al., 2018), and SOCS1 (Yang et al., 2020), which are potential targets of LRRC41 in methylation regulation of HCC. Additionally, KIAA1429 might interact with EGFR (Ye et al., 2016), HSPA8 (Khosla et al., 2019), and HSP90AA1 (Shi et al., 2020) to modulate methylation in HCC. As such, these underlying targets exhibited promising potential to act as critical regulators involving in the DNA methylation in HCC and further mediated tumorigenicity and progression.

### Landscape of Somatic Mutations in HCC

As summarized in the waterfall map, 327 out of 364 HCC patients had the somatic mutation altered, accounting for 89.84%. And we observed that TP53, CTNNB1, and TTN mutations are the top three mutated genes in HCC samples, and the frequency was 30, 25, and 24%, respectively (Supplementary Figure S11A). Missense mutations occupied an absolute position in the total mutation classification (Supplementary Figure S11Ba), and single-nucleotide polymorphism (SNP) accounted for more proportion than deletion (DEL) or insertion (INS, Supplementary Figures S11Bb,Be). Meanwhile, C > T had the highest frequency, 13933 times, in variant types of single-nucleotide variant (SNV) (Supplementary Figures S11Bc,D). Supplementary Figure S11Bd presents that the number of variants per sample and the median value of mutation variants were 71. Besides, the top 10 genetical variated genes were TP53, TTN, CTNNB1, MUC16, ALB, PCLO, MUC4, APOB, RYR2, and ABCA13 (Supplementary Figure S11Bf). The rainfall plot of the sample TCGA−UB−A7MB−01A−11D−A33Q−10 is presented in Supplementary Figure S11C. Each dot represents the SNV mutation type with corresponding color. To further elucidate the intrinsic connection between these genetic altered genes, the exclusive and co-occurrence correlations are presented in Supplementary Figure S11E. To further reveal the intrinsic connection of gene mutation status with DNA methylation, the top three mutated genes (TP53, TTN, and CTNNB1) were fetched for correlation analysis. The results showed that the TP53 mutation and CTNNB1 mutation were significantly higher in the KIAA1429 hypomethylation group (Figures 10G,H), but not TTN mutation (Figure 10F). However, there was no significant correlation of the LRRC41 methylation level with gene mutation (Figures 10C–E).

## Discussion

Hepatocellular carcinoma (HCC), well characterized with high morbidity, ranks fourth among tumor-caused deaths globally (1–3). Well characterized with genomic heterogeneity and genetic diversity, HCC patients presented high individual different clinical outcomes based on traditional classification (5, 6). Lacking practical clinical treatment, the overall survival probability of HCC patients remained very low. Therefore, it is necessary to exploit novel and reliable biomolecular indicators for prognosis prediction and clinical efficiency evaluation, contributing to novel insight into therapeutic response monitoring and clinical intervention of HCC.

DNA methylation, mediated in the gene transcription regulation and genome stability maintenance, is one of the most common types of inherited epigenetic modification. Aberrant changes in DNA methylation exist in multiple malignant tumor development (Yang et al., 2017), regulating the expression level of cancer-related genes and significantly affecting the progression of tumor. To further elucidate the intrinsic molecular mechanism of HCC progression, genetic indicators especially DNA CpG sites are critical (Xu et al., 2017; Zheng et al., 2018). Besides, clinical samples (i.e., body fluids) for the determination of the DNA methylation level can be obtained noninvasively from patients, providing a novel channel for early diagnosis, clinical management, and therapeutic targeting. Up to now, the potential role of DNA methylation sites in TIME and ICB therapy of HCC is still unclear.

Herein, this study was designed to uncover the prognostic predictive value and impact upon TIME characteristics and immunotherapy outcome of methylation sites in HCC. We analyzed the methylation information of HCC patients from the TCGA database through employing systematic bioinformatics analysis. Using consensus clustering, we identified seven HCC clusters based on their methylation data to better elucidate their clinical significance as well as biological role in progression of HCC.

Using univariate Cox regression and subsequent LASSO algorithm, we generated a two-gene risk signature consisting of LRRC41 and KIAA1429. In order to demonstrate its great prognostic accuracy, these results were validated in both the testing group and the external validation group. Besides, the prognostic value was demonstrated in another random grouping. The results showed that the risk signature could be an independent prognostic prediction factor using univariable and multivariable regression analyses. Furthermore, a robust quantitative nomogram plot including the risk score and clinical stage was constructed. GSEA analysis results suggested a potential biological molecular mechanism of risk signature in HCC progression *via* Wnt (Dai et al., 2019; Hu et al., 2019; Huynh et al., 2019; Jin et al., 2019; Li et al., 2019; Tan et al., 2019) and ERBB (Ni et al., 2020) signaling pathways and others. Besides, we demonstrated the prognostic risk signature remained a good prognosis predictive accuracy indicator when HCC cases subdivided into subgroups according to clinical features.

Based on published researches, we observed that some studies have revealed the correlation between DNA methylation with immunotherapy and immune infiltration, which could not be elucidated from the traditional RNA regulation viewpoint. Fietz S et al. examined CTLA4 promoter methylation for predicting objective response to anti-CTLA-4 immunotherapy in late-stage melanoma (Fietz et al., 2020). Nair V S et al. pointed out that T-cell exhaustion and immune checkpoint biomarkers were abnormally altered in tumor-infiltrating CD8^+^ and CD4^+^ T cells and tumor tissues in colorectal cancer (CRC) (Nair et al., 2020). Thus, we deduced the proportion of immune cells and level of immune infiltration in TIME were significantly correlated with gene expression and methylation. In summary of immune filtration results (i.e., CIBERSORT, ssGSEA, and TIMER), patients with high risk presented abundance of immune cells, which suggested the activated immune phenotype. However, the positive correlation of the risk score with immunotherapeutic target expression (i.e., PDCD1 and CTLA4) indicated that patients in the high-risk group might be more affected by immune checkpoint blockade–related pathways and suitable for immunotherapy to improve their poor prognosis. However, these results needed to be tested in *in vitro* or *in vivo* studies about the underlying molecular mechanism of immune response of HCC.

Being the encouraging and promising outcome of immune checkpoint blockade (ICB) therapy, immune checkpoint inhibitors have great influence upon clinical administration in anti-tumor therapy (Pitt et al., 2016; Llovet et al., 2018; Salik et al., 2020). Immune checkpoint inhibitor treatment has opened up a novel approach for clinical decision-making in HCC patients (Ng et al., 2020). However, HCC patients get relative little therapeutic efficiency after treating ICB therapy, and about 30% HCC patients obtained benefit from immune checkpoint inhibitor administration (Liu et al., 2020). Such biomarkers as tumor mutational burden and microsatellite instability were unreliable to accurately predict the clinical outcome of ICB therapy. It is, therefore, urgent to discover novel and promising factors that could predict response to ICB therapy for further individualized management and advanced precision immunotherapy (Nishino et al., 2017; Mushtaq et al., 2018; Ng et al., 2020). Multiple studies demonstrated that DNA methylation might act as a pivotal player in prediction of response to therapy (Yang et al., 2019; Li et al., 2020). In this study, we validated the DNA methylation–based risk score and potential hub targets (LRRC41 and KIAA1429) were significantly associated with expression level of ICB pivotal target genes (i.e., CTLA4). Besides, the as-constructed prognostic risk score significantly correlated with the ICB treatment target genes (i.e., CD274), suggesting patients with high risk might benefit from immunotherapy. These results indicated that the DNA methylation–based prognostic signature may provide novel insight into ICB therapy outcome prediction in HCC. Without ICB treatment–related data in the HCC cohort, we were unable to explore the correlation between ICB treatment response and risk score. Nevertheless, further experimental researches were required for our results at larger population and multiple centers.

Among DNA methylation–related genes in this prognostic signature, the player of LRRC41 in HCC has not been revealed in existing articles yet. Furthermore, we found the LRRC41 expression can independently affect the overall survival of HCC patients. LRRC41, a largely uncharacterized protein containing a leucine-rich repeat, serves as a pivotal modulator in the formation of cullin 3 (Cul3)–dependent ubiquitin ligase complexes (Schenková et al., 2012). Currently, the biological function of LRRC41 in tumors is still elusive. This study attempted to explore the prognostic predictive significance of LRRC41 and its potential functions in TIME and ICB treatment. We found that the LRRC41 expression level is significantly upregulated in HCC cells and is able to act as a good prognostic prediction factor in HCC. We also corroborated that both expression and methylation levels of LRRC41 had intimate correlation with immune infiltration (i.e., neutrophils) and immunotherapeutic targets (i.e., PDCD1). Additionally, the landscape of mutation status was delineated, and the correlation of methylation with gene mutation was explored. Nevertheless, the potential role of LRRC41 in HCC remains lacking, which needs further and deeper experimental exploration.

It is well established that inhibition of DNA methyltransferase will up-regulate immune signaling to reverse tumor-immune evasion, indicating the regulatory role of DNA methylation in programming the tumor immune microenvironment (Chiappinelli et al., 2015). The results of immune cell infiltration presented higher subpopulations of immune cells (i.e., CD8^+^ T cells) and active immunological signature (i.e., APC costimulation) in hypomethylation of LRRC41, indicating LRRC41 hypomethylation might contribute to anti-tumor immune response.

Relative to published articles that developed the novel prognostic predictive indicator in HCC, some superiorities of this study should be listed. Firstly, all HCC cases from the TCGA database and ICGC LIRI-JP dataset were included for systematic bioinformatic analysis, and the total specimen size was considerably large. Besides, we employed four methods (ssGSEA, CIBERSORT, TIMER, and single-cell RNA sequencing analysis) to explore the potential functions of DNA methylation in the context of TIME complexity and diversity, further contributing to ICB outcome prediction, which has not been reported before us. Furthermore, as far as we know, this research is the first aimed to explore the biological players of LRRC41 in HCC. Finally, multiple bioinformatics analyses were used for most data processing, all-image formation, and statistical analyses. All multiomics data were available from public datasets and R software (version 4.0.3) with corresponding packages having open access. However, the most notable limitation of this study is that further *in vivo* experimental study was not performed to validate our findings.

## Conclusion

In a word, we thoroughly analyzed the methylation landscape, prognostic prediction performance, and influence upon TIME and ICB therapy of DNA methylation in patients with HCC. The distinction of DNA CpG–related genes was a factor that was significantly correlated with overall survival and clinical features, indicating it may act as a pivotal player in the heterogeneity and complexity of tumor immune microenvironment. The systematic analysis of DNA methylation sites in tumor could strengthen our understanding of TIME characterization and facilitate personalized therapy administration. However, these results need to be further validated in subsequent *in vitro* and *in vivo* experimental and clinical researches focusing on HCC tumorigenesis, progression of biomolecular mechanisms, and potential player of these DNA methylation sites and corresponding genes.

## Data Availability

The datasets presented in this study can be found in online repositories. The names of the repository/repositories and accession number(s) can be found in the article/Supplementary Material.

## Glossary

ANOVA: analysis of variance
AUC: area under the curve
BCLC: Barcelona Clinic Liver Cancer
CD274: Also known as PD-L1
CDF: cumulative distribution function
CI: confidence interval
CpG: 5′-cytosine-phosphate-guanine-3′
CTLA‐4: cytotoxic T‐lymphocyte antigen 4
Cul3: cullin 3
DEL: deletion
DFS: disease-free survival
DMEM: Dulbecco’s minimum essential media
DNMT: DNA methyltransferase
FBS: fetal bovine serum
FDR: false discovery rate
FPKM: fragments per kilobase per million
GAPDH: glyceraldehyde-3-phosphate dehydrogenase
GO: gene ontology
GSEA: gene set enrichment analysis
GTEx: Genotype-Tissue Expression
HAVCR2: Also known as TIM-3
HCC: hepatocellular carcinoma
HR: hazard ratio
ICB: immune checkpoint blockade
ICGC: International Cancer Genome Consortium
IDO1: indoleamine 2,3‐dioxygenase 1
INS: insertion
KEGG: Kyoto Encyclopedia of Genes and Genomes
LASSO: least absolute shrinkage and selection operator
MAPK: mitogen-activated protein kinase
MSI: microsatellite instability
OS: overall survival
PAC: proportion of ambiguous clustering
PD‐1: programmed cell death 1
PD‐L1: programmed cell death-ligand 1
PD‐L2: programmed cell death-ligand 2
PDCD1: Also known as PD-1
PDCD1LG2: Also known as PD‐L2
qRT-PCR: quantitative real-time polymerase chain reaction
RNA: ribonucleic acid
ROC: receiver-operating characteristic
SAM: S-adenosyl-l-methionine
SNP: single-nucleotide polymorphism
SNV: single-nucleotide variant
ssGSEA: single-sample gene set enrichment analysis
TCGA: The Cancer Genome Atlas
TICs: tumor‐infiltrating immune cells
TIM‐3: T‐cell immunoglobulin domain and mucin domain–containing molecule‐3
TIME: tumor immune microenvironment
TIMER: tumor immune estimation resource
TMB: tumor mutation burden
TNM: topography lymph node metastasis
TPM: transcript per million
UCSC: University of California, Santa Cruz
