# Supplementary figures and images for: Immunological Significance of Prognostic DNA Methylation Sites in Hepatocellular Carcinoma
Source: Front Mol Biosci. 2021 May 26;8:683240. doi: 10.3389/fmolb.2021.683240 (PMC8187884; doi:10.3389/fmolb.2021.683240)

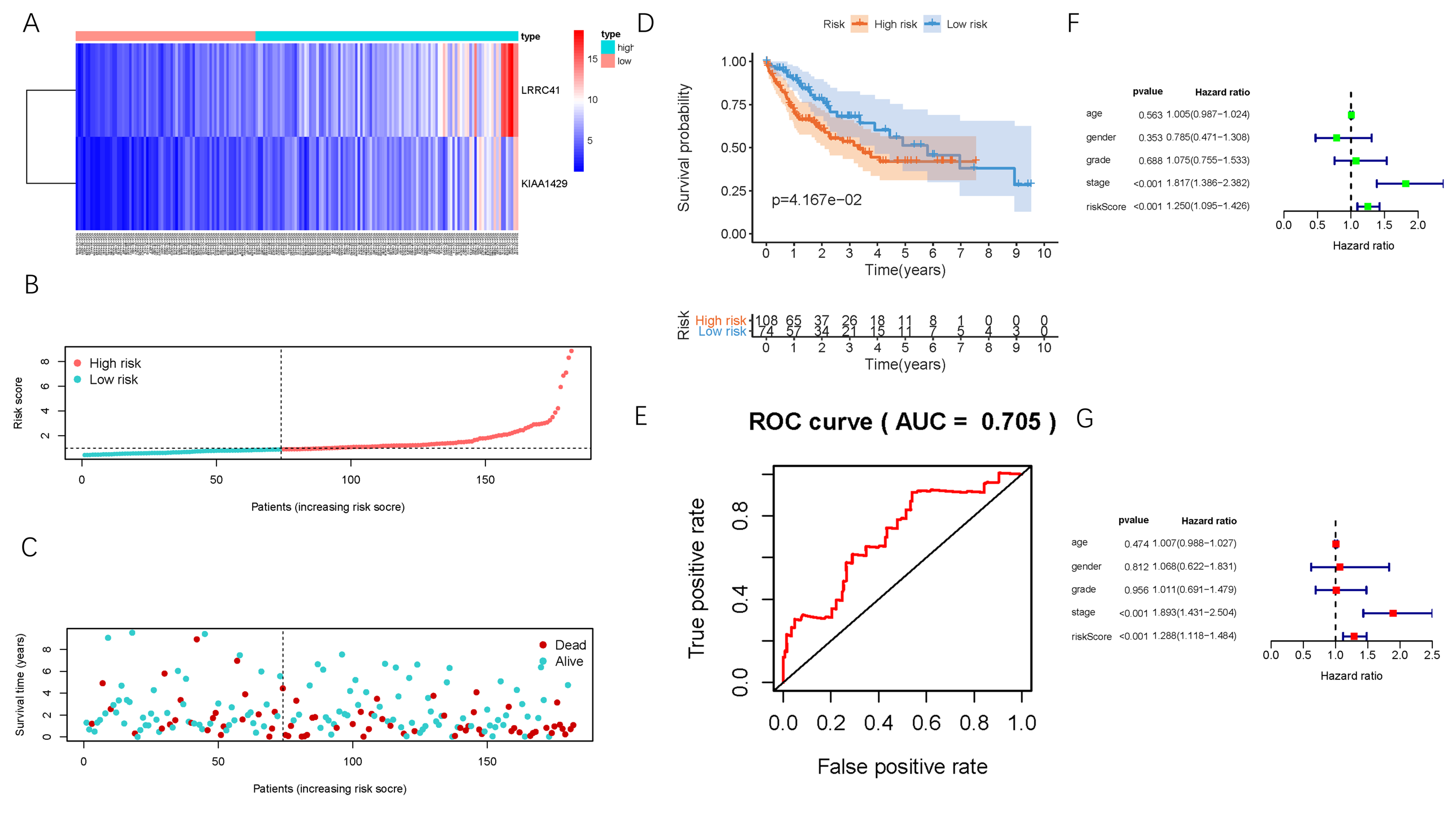

Supplement: Supplementary file 3 [file Image6.TIF]

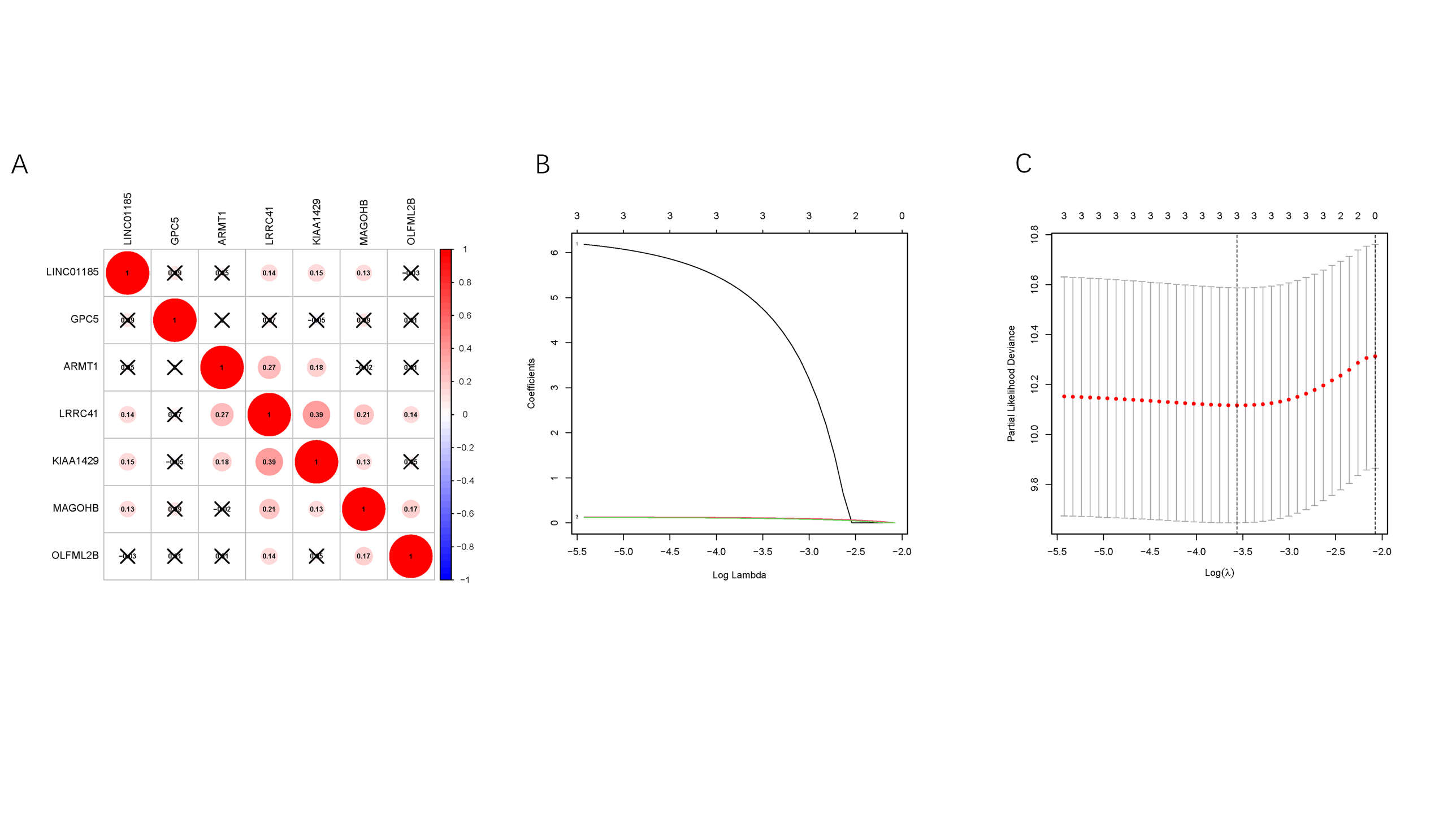

Supplement: Supplementary file 5 [file Image3.TIF]

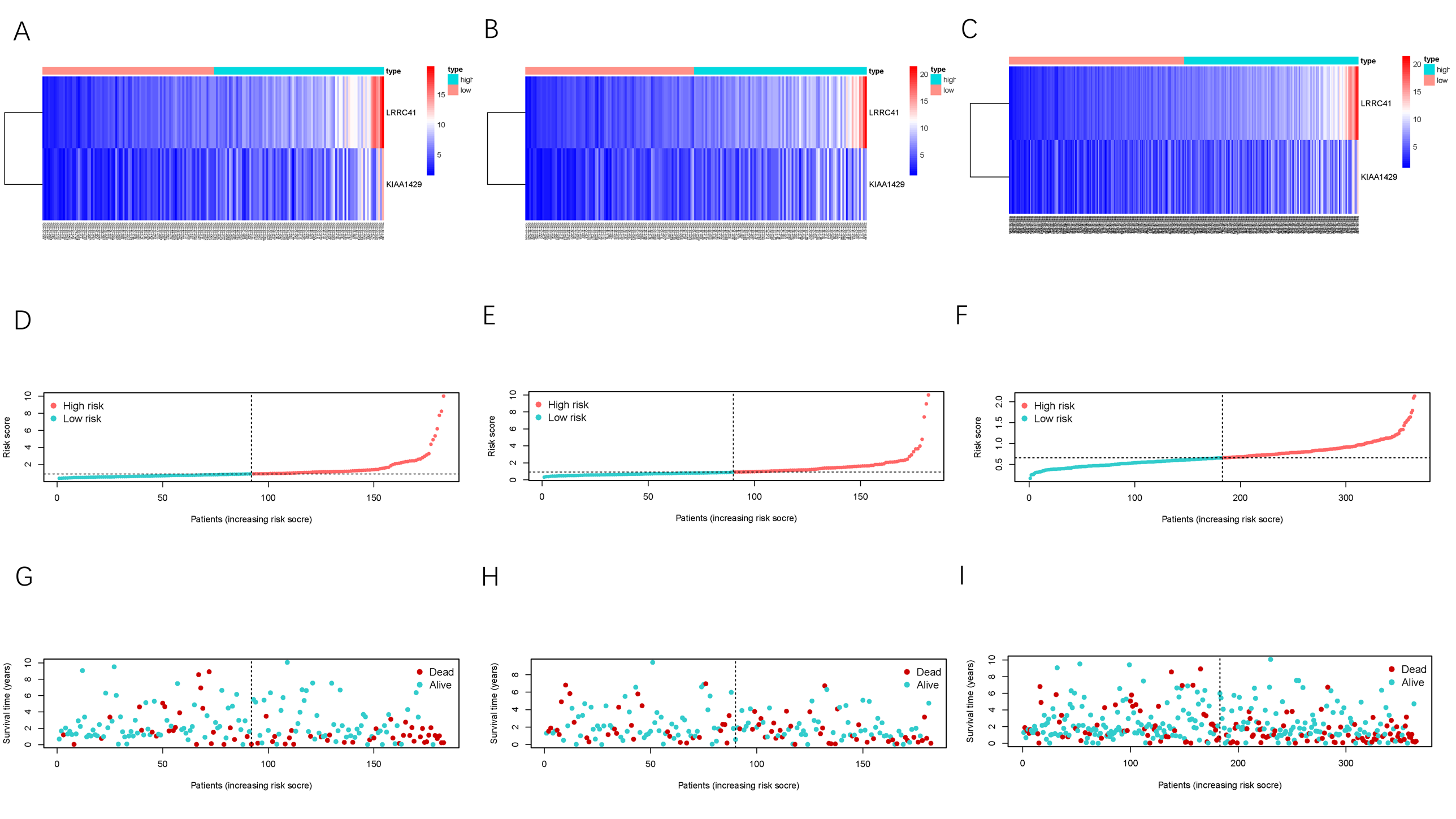

Supplement: Supplementary file 6 [file Image4.TIF]

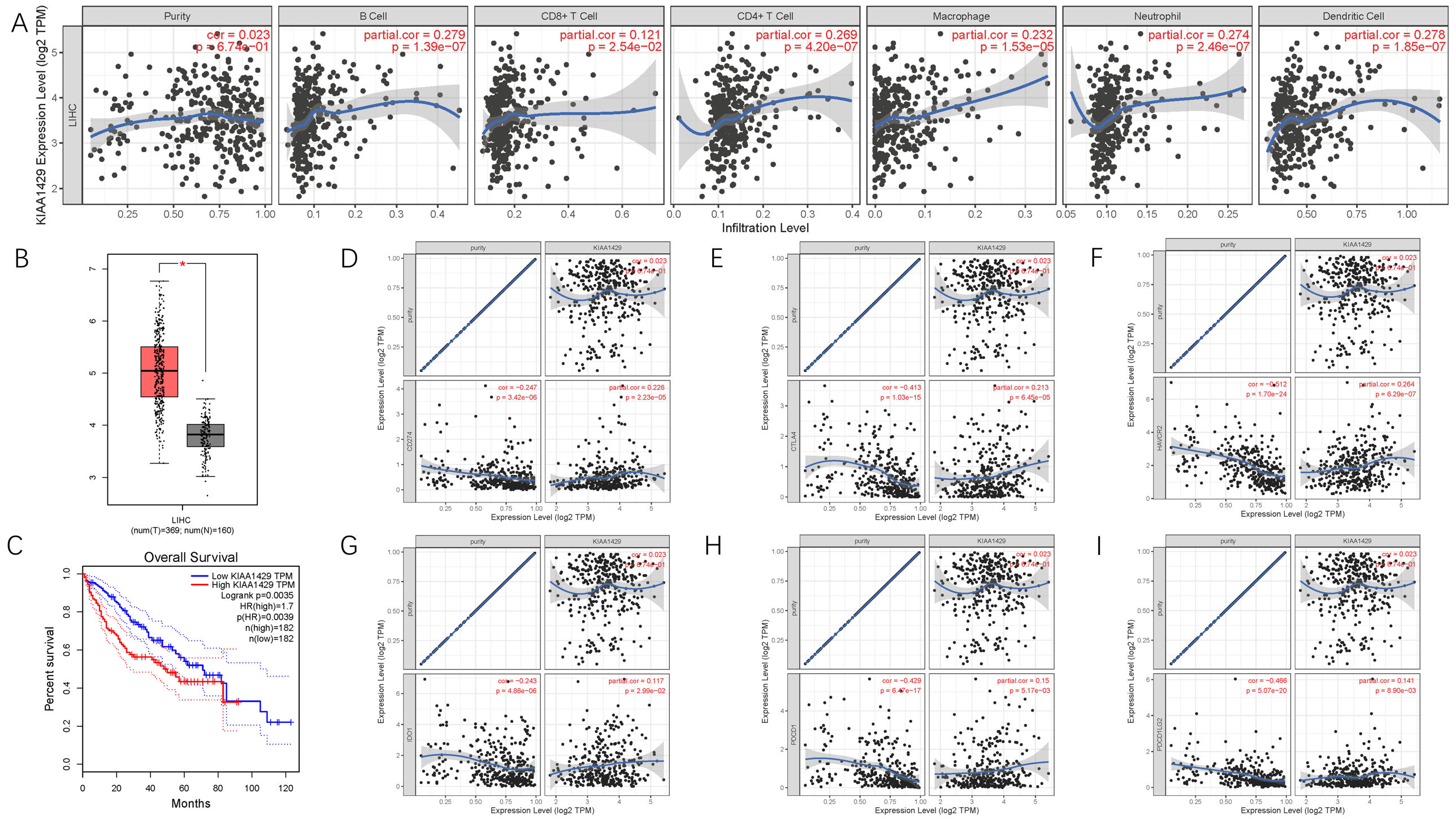

Supplement: Supplementary file 7 [file Image9.TIF]

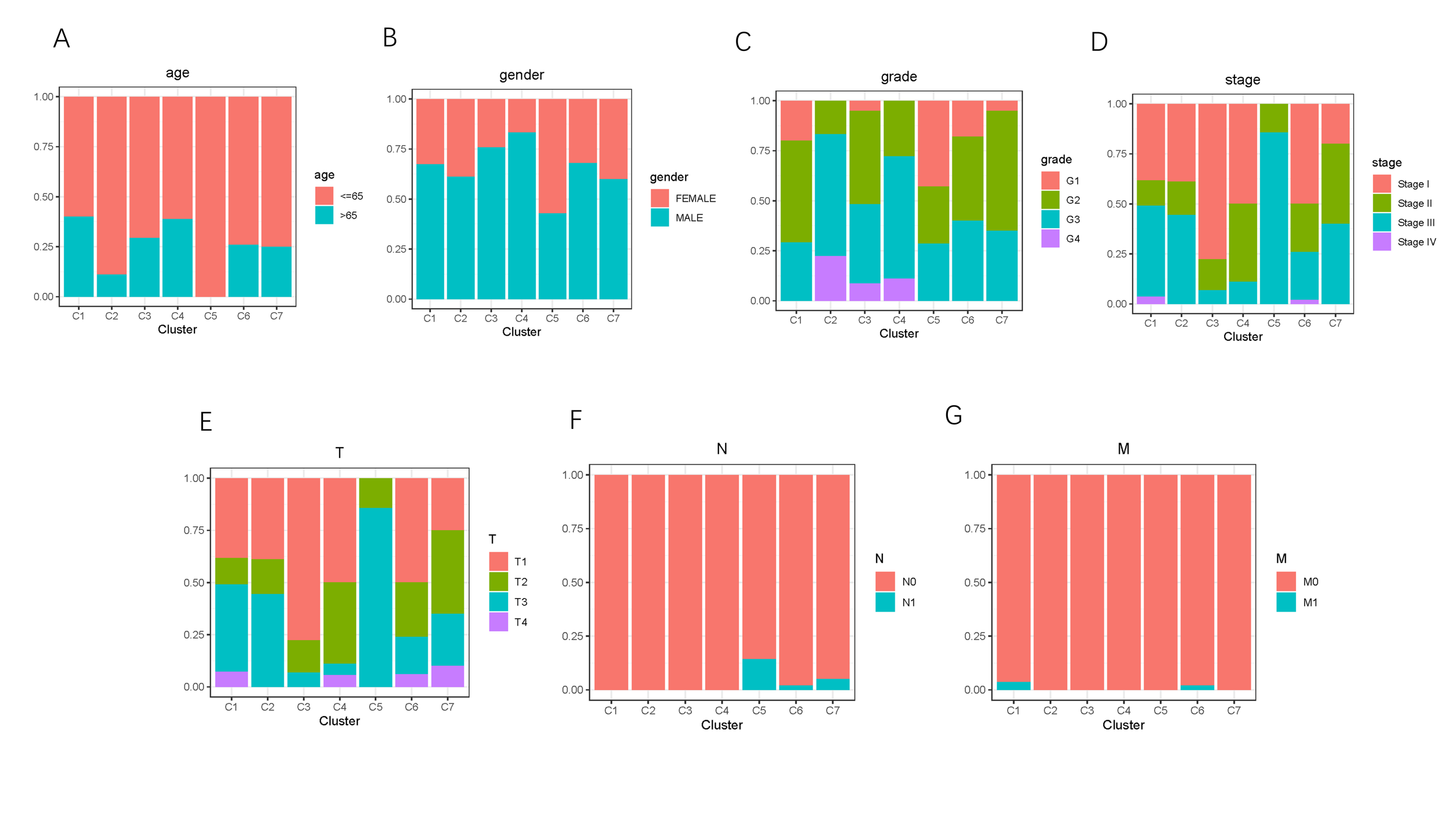

Supplement: Supplementary file 8 [file Image2.TIF]

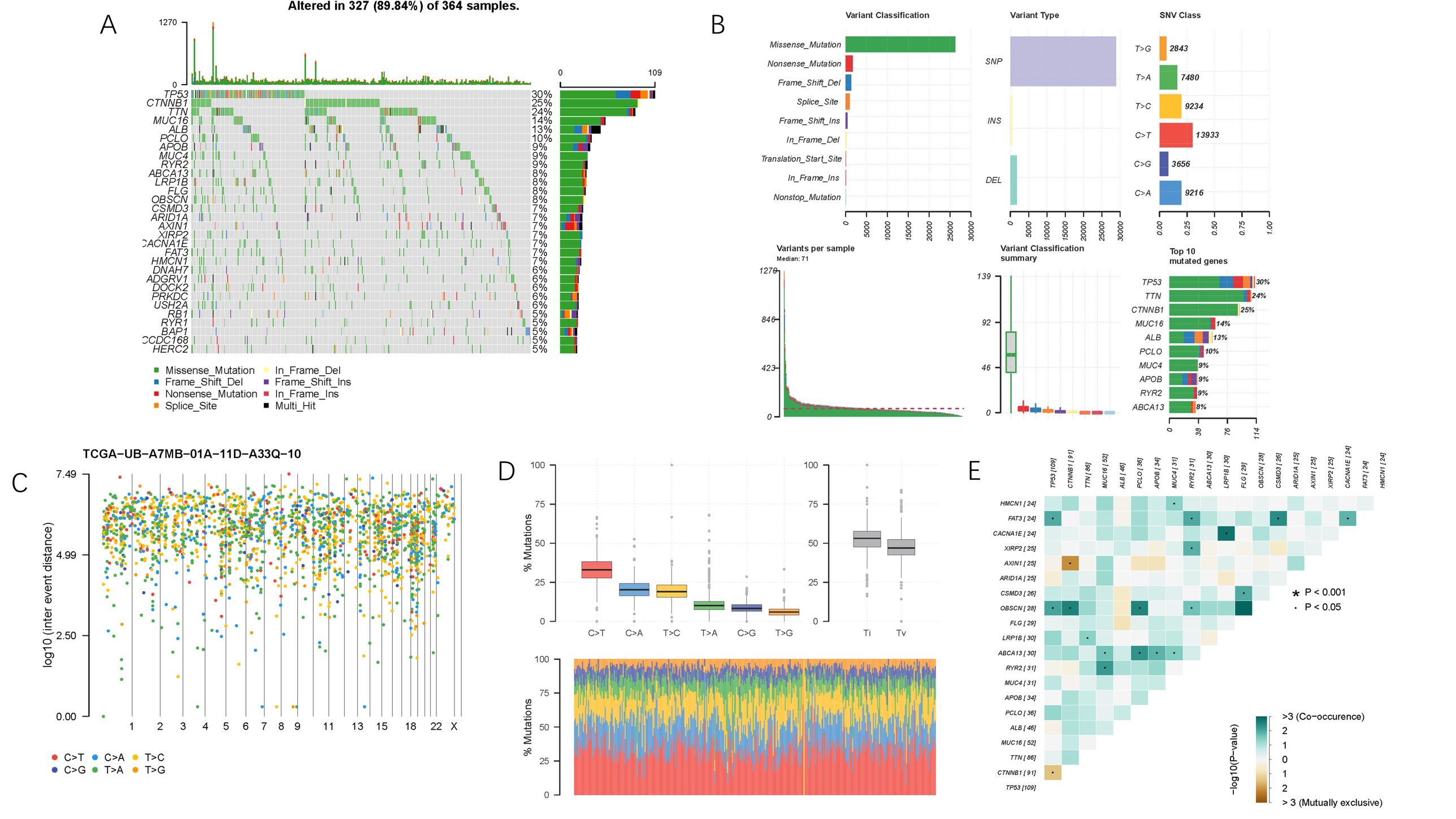

Supplement: Supplementary file 9 [file Image11.TIF]

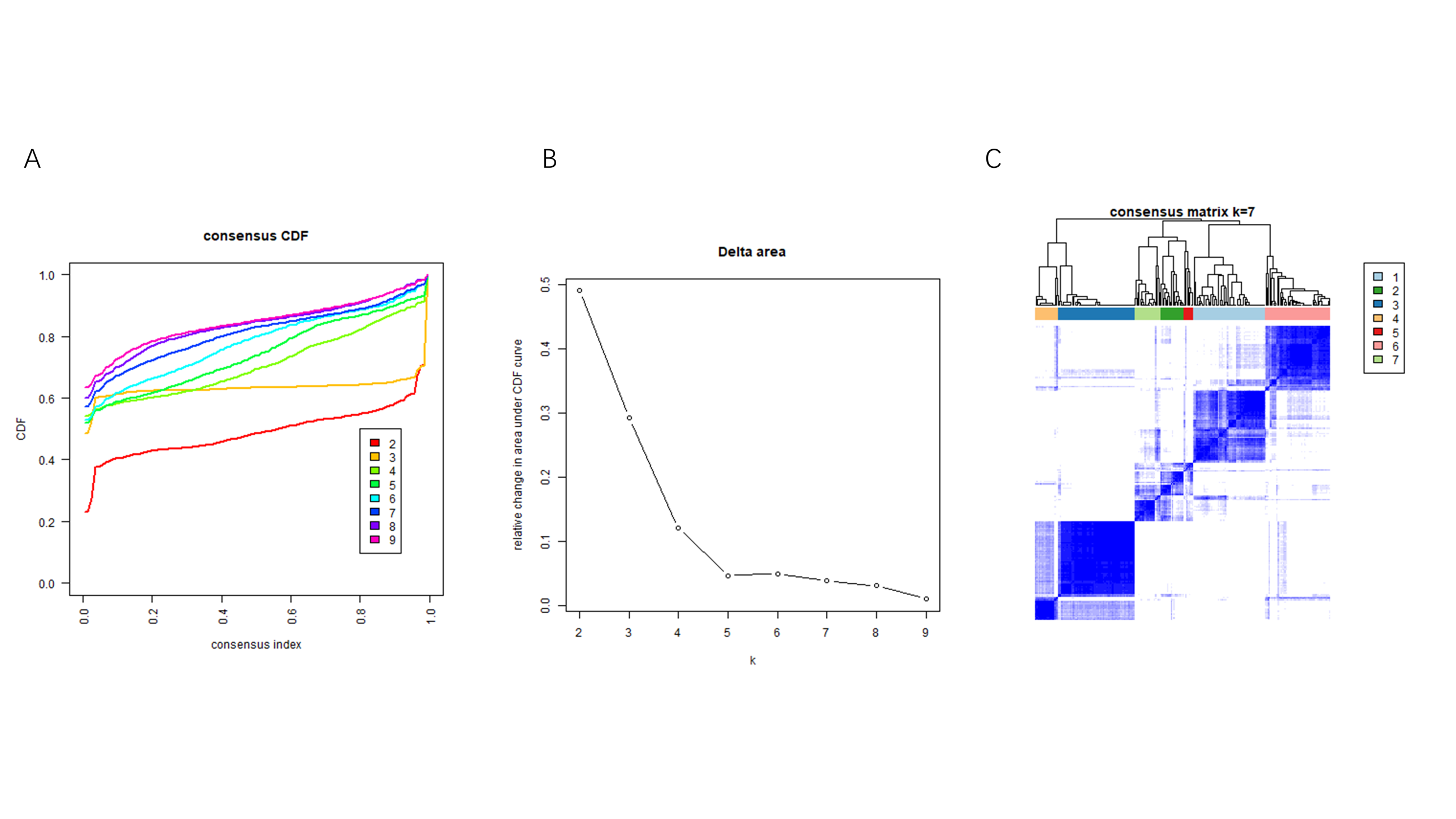

Supplement: Supplementary file 10 [file Image1.TIF]

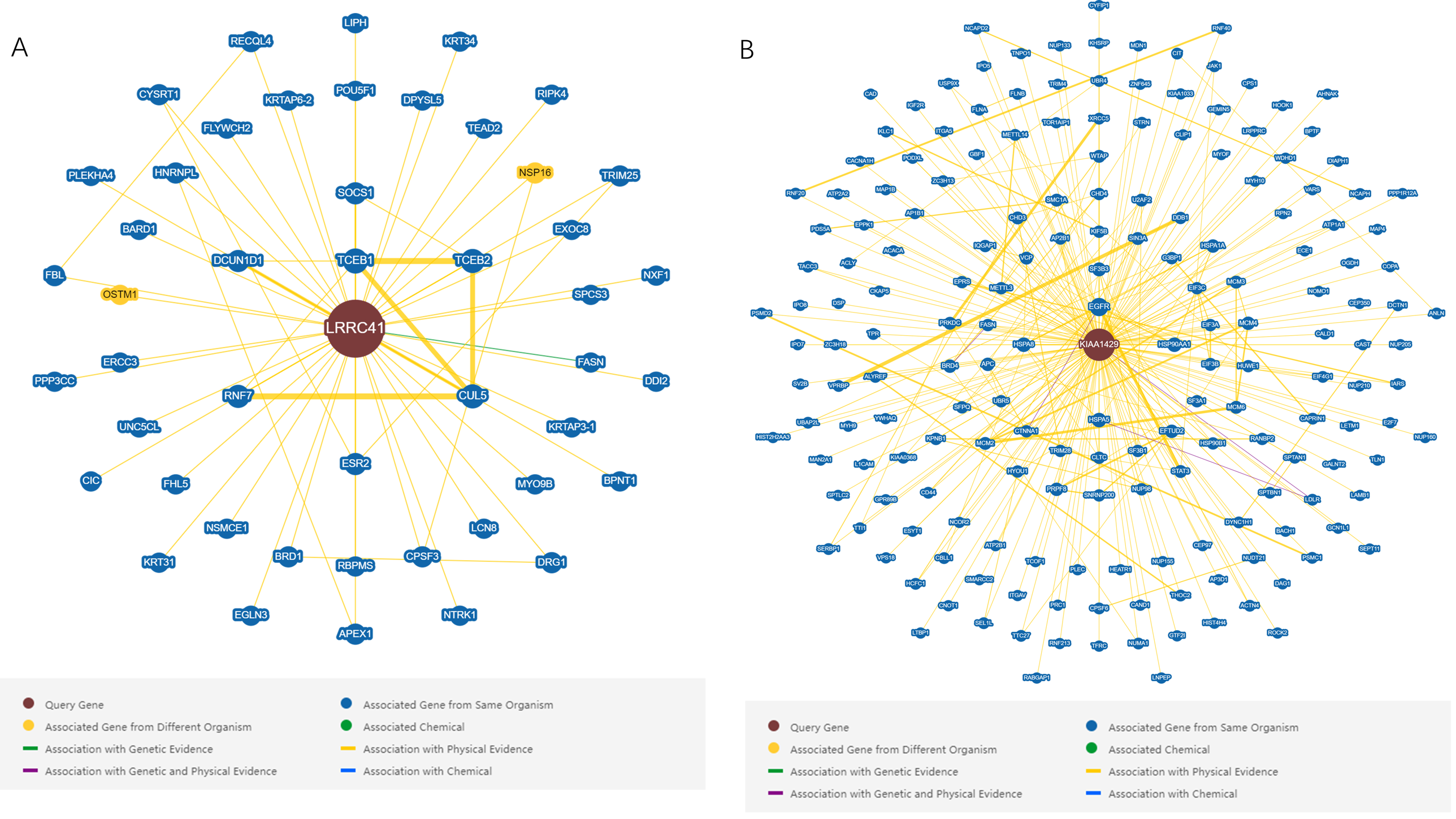

Supplement: Supplementary file 11 [file Image10.TIF]

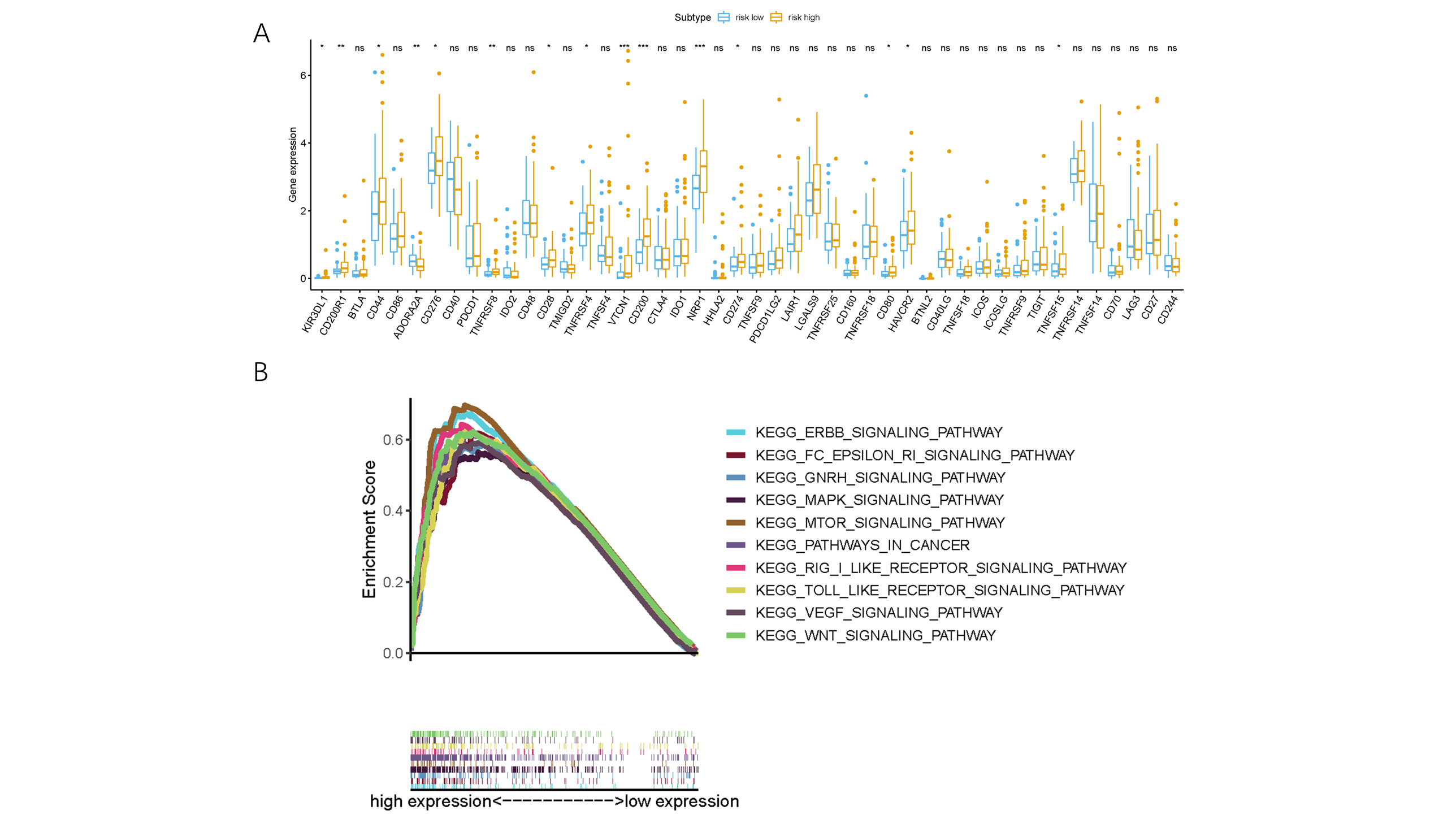

Supplement: Supplementary file 12 [file Image7.TIF]

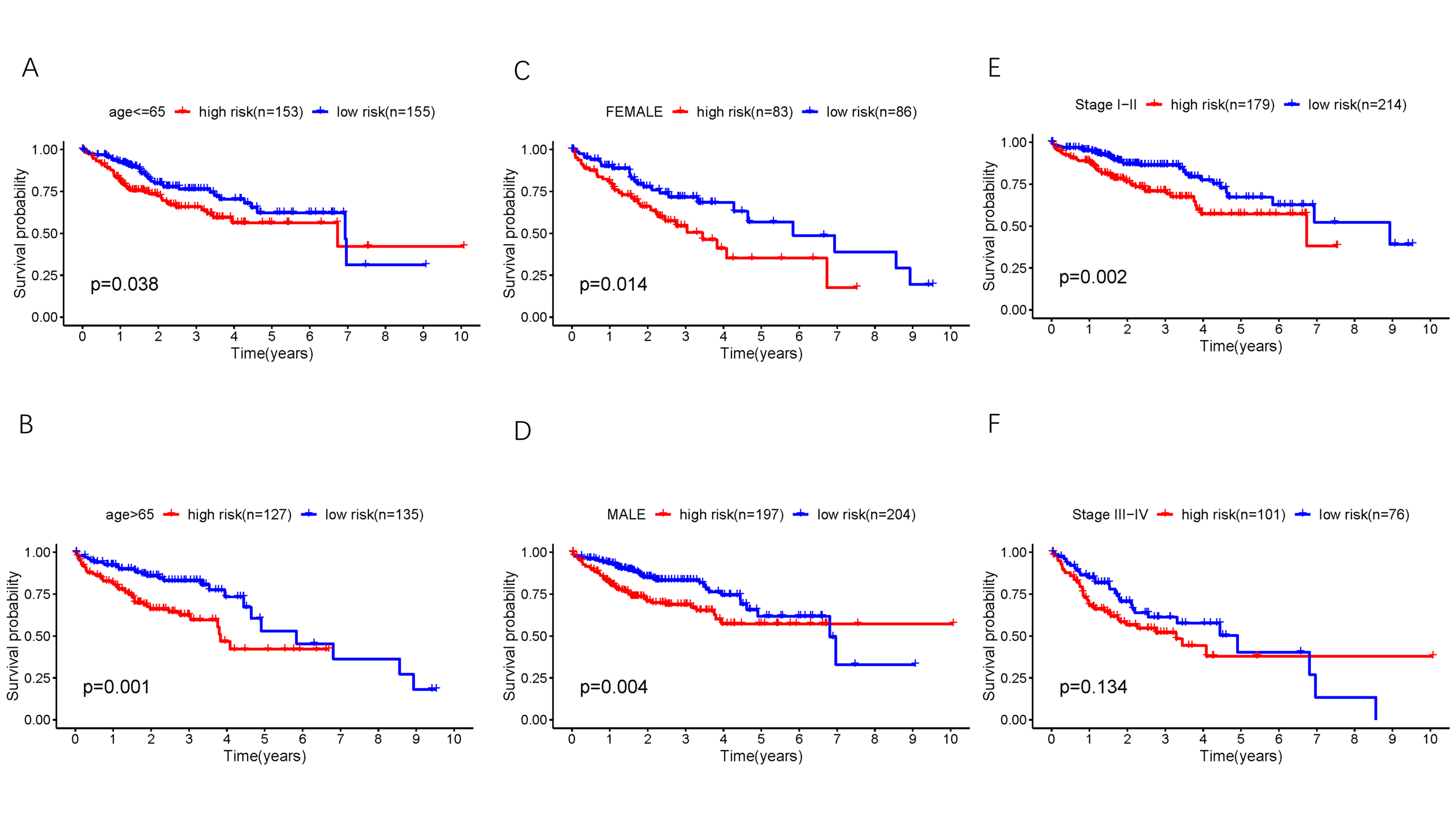

Supplement: Supplementary file 17 [file Image8.TIF]

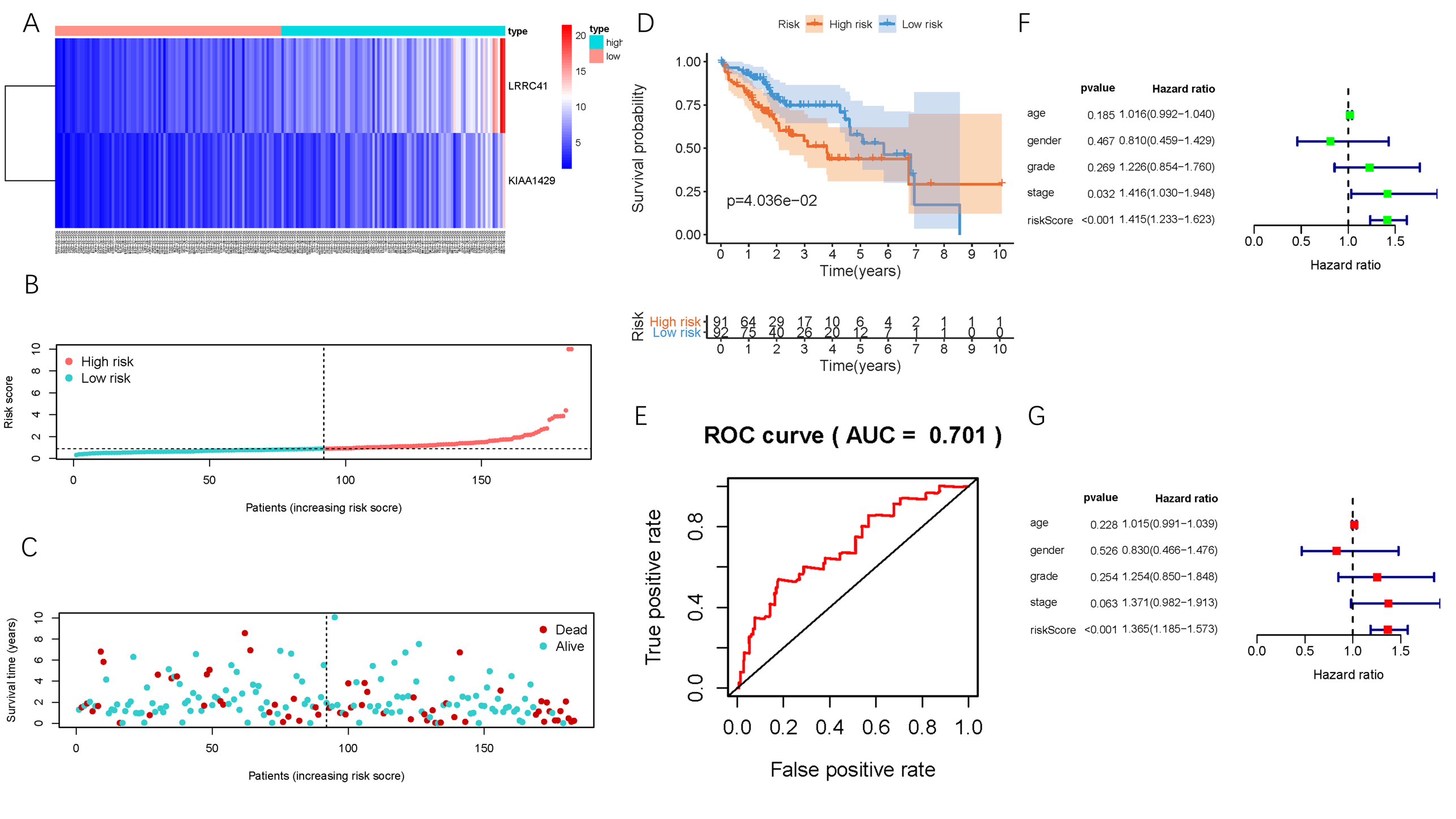

Supplement: Supplementary file 20 [file Image5.TIF]
